# Pyruvate Oxidase as a Critical Link between Metabolism and Capsule Biosynthesis in *Streptococcus pneumoniae*

**DOI:** 10.1371/journal.ppat.1005951

**Published:** 2016-10-19

**Authors:** Haley Echlin, Matthew W. Frank, Amy Iverson, Ti-Cheng Chang, Michael D. L. Johnson, Charles O. Rock, Jason W. Rosch

**Affiliations:** 1 Department of Infectious Diseases, St Jude Children’s Research Hospital, Memphis, Tennessee, United States of America; 2 Department of Computational Biology, St Jude Children’s Research Hospital, Memphis, Tennessee, United States of America; University of Adelaide, AUSTRALIA

## Abstract

The pneumococcus is one of the most prodigious producers of hydrogen peroxide amongst bacterial pathogens. Hydrogen peroxide production by the pneumococcus has been implicated in antibiotic synergism, competition between other bacterial colonizers of the nasopharynx, and damage to epithelial cells. However, the role during invasive disease has been less clear with mutants defective in hydrogen peroxide production demonstrating both attenuation and heightened invasive disease capacity depending upon strain and serotype background. This work resolves these conflicting observations by demonstrating that the main hydrogen peroxide producing enzyme of the pneumococcus, SpxB, is required for capsule formation in a strain dependent manner. Capsule production by strains harboring capsules with acetylated sugars was dependent upon the presence of *spxB* while capsule production in serotypes lacking such linkages were not. The *spxB* mutant had significantly lower steady-state cellular levels of acetyl-CoA, suggesting that loss of capsule arises from dysregulation of this intermediary metabolite. This conclusion is corroborated by deletion of *pdhC*, which also resulted in lower steady-state acetyl-CoA levels and phenocopied the capsule expression profile of the *spxB* mutant. Capsule and acetyl-CoA levels were restored in the *spxB* and *lctO* (lactate oxidase) double mutant, supporting the connection between central metabolism and capsule formation. Taken together, these data show that the defect in pathogenesis in the *spxB* mutant is due to a metabolic imbalance that attenuates capsule formation and not to reduced hydrogen peroxide formation.

## Author Summary

The pneumococcus polysaccharide capsule is one of the most critical virulence determinants produced by this major human pathogen. The pneumococcus also produces prodigious amounts of hydrogen peroxide via the enzymatic reaction catalyzed by pyruvate oxidase, SpxB. Deletion of *spxB* resulted in the loss of surface polysaccharide capsule production in a serotype dependent manner with a mirrored effect on the virulence of the mutants. We observed that deletion of *spxB* reduced the steady-state levels of acetyl-CoA, a key metabolic intermediate in peptidoglycan, fatty acid biosynthesis, and in capsule biosynthesis in a subset of serotypes. These data suggest that the defect in capsule production was due to altered metabolism that results in reduced acetyl-CoA availability. Corroborating these data, we found that capsule biosynthesis was impaired upon loss of PDHC, an additional metabolic enzyme that generates acetyl-CoA. These data reveal a critical link between pneumococcal metabolism and capsule biosynthesis as well as provide a striking example of how a virulence gene can have a differential contribution to pathogenesis dependent upon strain background.

## Introduction

*Streptococcus pneumoniae* is a major human pathogen whose disease manifestations range from sinusitis and otitis media to pneumonia, bacteremia, and meningitis. The pneumococcus produces a number of important virulence factors, including the polysaccharide capsule, one of the most widely distributed virulence factors and the target of current vaccines. In addition to the capsule and a variety of other surface-associated and secreted virulence factors, the pneumococcus is also a prodigious producer of hydrogen peroxide, generating millimolar amounts as a product of central metabolism. The levels of hydrogen peroxide produced are sufficient to mediate bactericidal activity of other bacterial species upon co-culture and is thought to be important for competing against other bacterial species in the relatively resource restricted niche of the nasal passages [1–3]. In addition, the levels of hydrogen peroxide produced are sufficient to mediate DNA damage and subsequent apoptosis in lung cells as well as driving expression of multiple inflammatory genes, underscoring the potential capacity as a mechanism for tissue damage during infection [4–6].

While hydrogen peroxide damages other cells, the pneumococcus has to contend with the copious amounts of endogenous hydrogen peroxide produced and resist oxidative stress produced during normal metabolism [7, 8]. Oxidant damage alone has the potential to inhibit bacterial growth but controversial evidence indicates that multiple classes of antibiotics induce cellular redox stress, which could potentially synergize with the antibiotics for lethal effect [9–15]. Recent evidence has indicated that loss of SpxB, pyruvate oxidase, in pneumococcus renders the cells more resistant to fluoroquinolone antibiotics [16]. Hence, manipulation of the hydrogen peroxide producing capacity of the pneumococcus may be expected to affect susceptibility to host and exogenous antimicrobial peptides, and, consequently, virulence.

The direct role that SpxB plays in pneumococcal virulence during infection is debated. During active infection, hyper-virulent mutants with defective *spxB* can be generated in the serotype 1 background, yet these strains have a fitness tradeoff during colonization [17]. Increase in capsule production upon *spxB* deletion has also been observed in type 2 background, suggesting increased virulence with loss of pyruvate oxidase activity [18]. Other groups have found that deletion of *spxB* results in a significant delay in time to death in rodent models of infection [19, 20]. Together, these studies indicate that the contribution of *spxB* may vary considerably between pneumococcal serotypes and that the mechanism underlying this differential contribution to pathogenesis remains poorly characterized. The main focus of studies of pyruvate oxidase, SpxB, has been the generation of hydrogen peroxide, rather than the metabolic consequences of perturbing this pathway.

**Fig 1:**
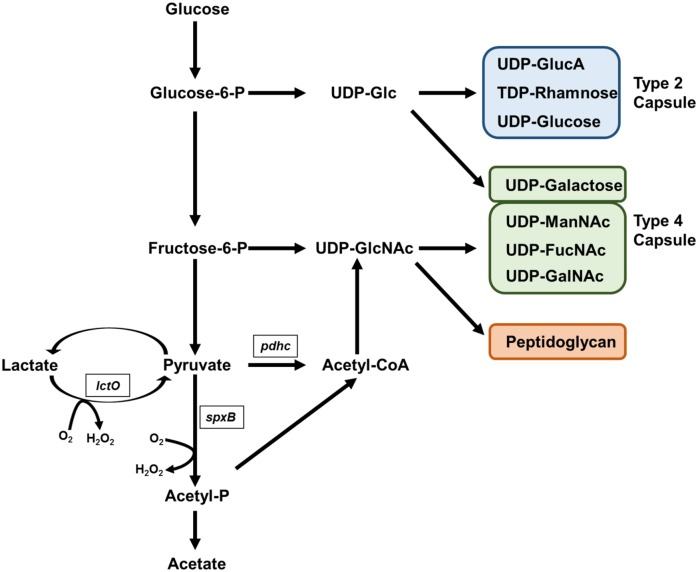
Model of glucose metabolism in *S*. *pneumoniae*.

The pneumococcal polysaccharide capsule is essential for invasive disease but is an energetically expensive virulence factor to produce, as the carbohydrates utilized could otherwise support energy production through glycolysis [21]. SpxB is directly linked to glycolysis in that it converts pyruvate to acetyl-phosphate, which can be converted to acetate via acetate kinase or to acetyl-CoA via phosphate acetyltransferase (Fig 1). Hydrogen peroxide is one of the products of the enzymatic activities of SpxB. Downstream effects of deletion of *spxB* results in altered levels of a number of key metabolites, including both ATP and acetyl-phosphate [22, 23]. An additional metabolic pathway for generation of hydrogen peroxide also exists in the streptococci, namely the activity of lactate oxidase, LctO, which converts lactate into pyruvate, the substrate for SpxB [24, 25]. Little is understood about how perturbation of these key metabolic pathways affects capsule production, and thus virulence.

In this manuscript, we evaluated the role of hydrogen peroxide production in pneumococcal susceptibility to antimicrobial peptide, LL-37. Surprisingly, the *spxB* mutant displayed enhanced resistance to LL-37 whereas the double *spxB* and *lctO* mutant had similar sensitivity to wild type, suggesting that LL-37 resistance occurred via a hydrogen peroxide independent manner. Upon further study, we discovered that deletion of *spxB* in a serotype 4 background abrogated capsule biosynthesis, a phenotype that was rescued in the double mutant. The virulence potential of the strains mirrored the capsule phenotype, with the *spxB* mutant becoming highly attenuated for invasive disease whereas the double mutant retained pathogenicity. These data indicate that loss of virulence occurs in a hydrogen peroxide dependent manner. The loss of capsule production in the *spxB* mutant was found to be restricted to a subset of capsule types—to those with acetylated sugars in their capsule. This strain dependent loss of capsule may be explained by altered metabolism which leads to dysregulation of acetyl-CoA, as we found greatly reduced levels of acetyl-CoA in the *spxB* mutant in the type 4 background. Loss of the pyruvate dehydrogenase complex, an additional metabolic pathway that generates acetyl-CoA, also reduced capsule biosynthesis. These data indicate that the detriment to virulence upon loss of SpxB can be linked primarily to central metabolism and not to hydrogen peroxide production. This suggests that central metabolic processes can influence the capacity of the pneumococcus to synthesize the polysaccharide capsule and provide a link between central metabolism and virulence in this major human pathogen.

## Results

### Sensitivity of Hydrogen Peroxide Mutants to Antimicrobial Peptide LL-37

To ascertain whether reduced hydrogen peroxide production by the pneumococcus results in altered sensitivity to antimicrobials, stable deletions in *spxB* and *lctO* were generated individually as well as together to make a double mutant in two different pneumococcal serotypes—TIGR4 (type 4) and D39 (type 2). The loss of hydrogen peroxide production in each of the mutants was assessed by two methods (S1 Fig). In agreement with previous studies [19, 20, 22, 26], deletion of *spxB* resulted in significantly reduced hydrogen peroxide production, to approximately 20% of levels produced by wild type. The *lctO* mutant demonstrated a more modest reduction (50%) while the double mutant had only background levels of hydrogen peroxide production (5%). This pattern was observed in both the TIGR4 and D39 backgrounds. The loss of transcript in each of the mutants was confirmed using qRT-PCR (S1 Table).

**Fig 2:**
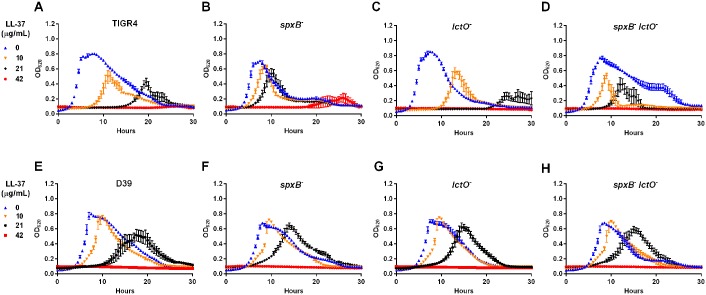
Deletion of *spxB* in TIGR4, but not in D39, demonstrates resistance to LL-37 killing. The growth of cells in increasing concentration of LL-37 (0, 10, 21, and 42 μg/ mL) was measured. Strains include those in type 4 background—TIGR4 (**A**) and the *spxB* (**B**), *lctO* (**C**), and double *spxB-lctO* (**D**) mutants—and those in type 2 background—D39 (**E**) and the *spxB* (**F**), *lctO* (**G**), and double *spxB-lctO* (**H**) mutants.

To determine whether the mutant strains displayed differential sensitivity to antimicrobials, we tracked the growth of the mutants in increasing concentrations of the antimicrobial peptide LL-37, which has been shown to induce oxidative stress in *E*. *coli* [15]. If sensitivity to LL-37 mirrors hydrogen peroxide production, those strains producing less hydrogen peroxide—the *spxB* and double mutants—would be more resistant. Compared to the parental TIGR4 strain (Fig 2A), the *spxB* mutant displayed heightened resistance to LL-37 (Fig 2B) by maintaining growth (albeit slightly slower) in LL-37 concentrations up to 21 μg/mL. The *lctO* mutant (Fig 2C) had similar sensitivity to the parental TIGR4 strain. Interestingly, the double mutant (Fig 2D) grew slower and had less overall density in 10 and 21 μg/mL LL-37 compared to the *spxB* mutant, suggesting that the double mutant, which produces minimal levels of hydrogen peroxide, is more sensitive to LL-37 than the *spxB* mutant. We next repeated this experiment with mutants generated in the serotype 2 background D39. No significant alterations in sensitivity were observed in any of the mutant strains (Fig 2E–2H). As the *spxB* mutant in both backgrounds have similarly reduced levels of hydrogen peroxide production, these results indicate that the heightened resistance of the *spxB* mutant to LL-37 was strain-dependent and was not solely due to reduced hydrogen peroxide production. The observed resistance of the *spxB* mutant to LL-37 may be due to an intrinsic ability of the *spxB* mutant to resist oxidative stress. To test this possibility, we measured gene expression changes in three oxidative stress response genes (*sodA*, *tpxD*, and *ertX1*) in the presence or absence of LL-37 in the respective strain backgrounds. For all three genes, there was no significant differences in transcript abundance between wild type and the *spxB* mutant with or without LL-37 (S2 Table), suggesting that the resistance of the *spxB* mutant is not due to altered transcriptional response to oxidative stress. Interestingly, in the double mutant, the intracellular oxidative response genes (*sodA* and *tpxD*) have reduced transcripts while the extracellular response gene (*ertX1*) has elevated transcript compared the wild type.

### Role of Pyruvate Oxidase in Capsule Biosynthesis

**Fig 3:**
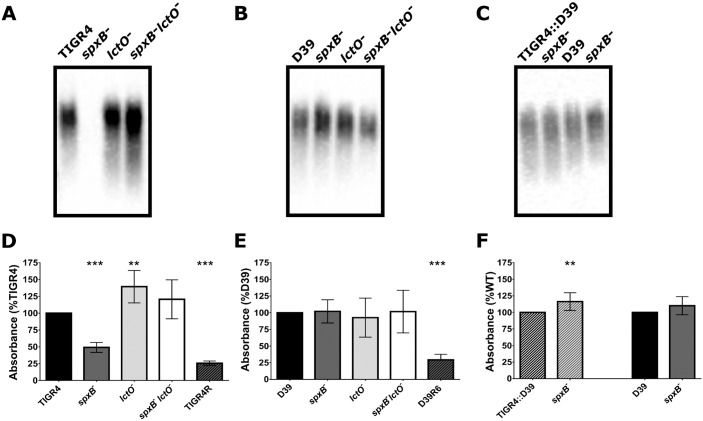
Capsule production is reduced in the TIGR4 *spxB* mutant. Capsule production was measured in two methods. In the first, cell lysates from wild type and the *spxB*, *lctO*, and double *spxB/lctO* mutants in either TIGR4 (**A**) or D39 (**B**) backgrounds and the wild type and *spxB* mutant of the capsule swapped strain (TIGR4::D39) (**C**) were subjected to capsule blotting. In the second, immunoreactivity against capsule was determined using whole-cell bacterial ELISA for the TIGR4 (**D**), D39 (**E**), and TIGR4::D39 (**F**) strains. TIGR4R and D39 R6, acapsular variants, were included as a negative control (**D**, **E**). Capsule immunoreactivity was normalized to immunoreactivity to LytA, a cell surface protein, and then plotted as percentage of wild type. Mutants were compared to the wild type using unpaired parametric t-test; ** p = 0.01–0.001, *** p<0.001.

Recently, it has been demonstrated that loss of the polysaccharide capsule on the pneumococcal surface renders the bacterium more resistant to LL-37 [27]. One possible mechanism for the aforementioned observations then is that deletion of *spxB* results in downstream effects on capsule biosynthesis. As such, we next sought to determine if there were any differences in capsule production in the mutants. Using the capsule blot method and serotype-specific antibodies, we could not detect capsule in the TIGR4 *spxB* mutant (Fig 3A), whereas the *lctO* mutant maintained wild type levels of capsule. Interestingly, the double mutant, which was generated by introduction of the *lctO* mutation into the *spxB* mutant background, resulted in the restoration of the capsule to wild type levels. Furthermore, complementation of the double mutant with *lctO* expressed from a plasmid resulted in loss of capsule from this formerly encapsulated strain (S2 Fig), effectively recapitulating the phenotype observed in the *spxB* mutant. In the D39 background, however, there was no discernible effect of any of the mutations on capsule production (Fig 3B). These data suggest that deletion of *spxB* affects capsule production in either a strain specific or capsule specific manner. To determine this, we generated capsule swap strains whereby the type 4 capsule locus of TIGR4 was replaced with the type 2 capsule locus of D39, referred as TIGR4::D39, and subsequently deleted *spxB* (Fig 3C). Capsule production was observed in the *spxB* mutant in the capsule swapped strain, similar to that in the D39 background. Because the capsule swapped strain maintains the TIGR4 background while altering the capsule type, this result indicates that loss of capsule in the *spxB* mutant is capsule type specific.

We next sought to confirm the loss of capsule in the TIGR4 *spxB* mutant. First, we performed whole cell ELISA using serotype-specific antibodies. While the *spxB* mutant had greatly reduced amounts of immunoreactivity, both the *lctO* and the double mutants had equivalent or slightly greater levels of capsule production compared to the wild type in the TIGR4 background (Fig 3D). Of note, there is some residual nonspecific binding as the acapsular TIGR4R strain exhibits about half of the reactivity of the *spxB* mutant. In D39, the *spxB* mutant in D39 retained capsule production in comparison to the parental strain, similar to the *lctO* and double mutants (Fig 3E). Supporting this finding, in a previous study, the *spxB* mutant maintained capsule production in the D39 background [18]. The *spxB* mutant in the capsule swapped strain demonstrates slightly more capsule production than the wild type (Fig 3F).

**Fig 4:**
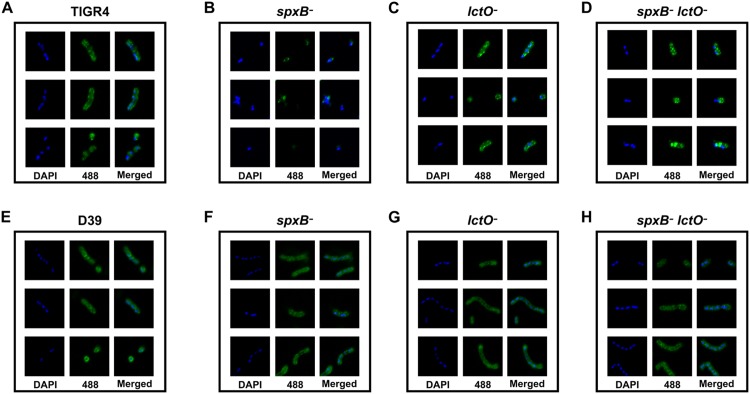
Fluorescence against surface capsule is greatly diminished in the TIGR4 *spxB* mutant. Capsule production was detected using fluorescence microscopy using primary antibodies against type 2 or 4 capsules with a fluorescent secondary (green) and DAPI stain to detect DNA (blue). Three representative images of TIGR4 (**A**) and the *spxB* (**B**), *lctO* (**C**), and double *spxB lctO* (**D**) mutants in the TIGR4 background and D39 (**E**) and the *spxB* (**F**), *lctO* (**G**), and double *spxB lctO* (**H**) mutants in the D39 background are shown.

As a confirmatory approach, we further assessed capsule production using fluorescence microscopy. While fluorescence against capsule could be observed surrounding the DAPI signal in the TIGR4 wild type (Fig 4A) and the *lctO* mutant (Fig 4C), very minor if any fluorescence against capsule was detected in the *spxB* mutant (Fig 4B). Interestingly, the detectable capsule fluorescence in the double mutant (Fig 4D) did not completely encompass the cellular surface, suggesting that the distribution of capsule is not as complete in the double mutant as in the wild type. In the D39 background (Fig 4E–4H), the *spxB* mutant, similar to the other mutants, displayed wild type levels and patterns of fluorescence against capsule.

**Fig 5:**
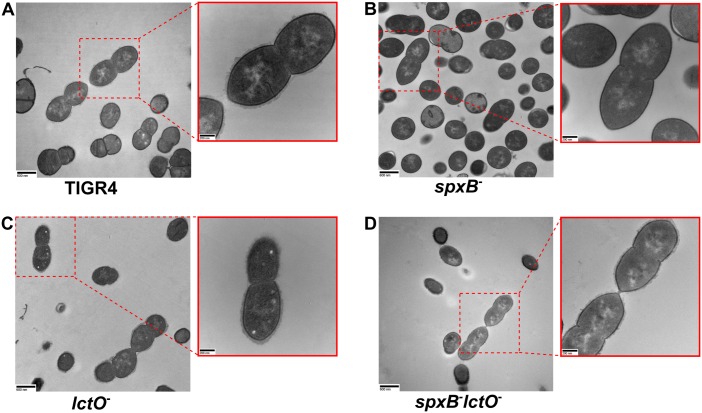
Capsule on the cell surface is undetectable in the TIGR4 *spxB* mutant. Cell surface structures of TIGR4 (**A**) and the *spxB* (**B**), *lctO* (**C**), and double *spxB lctO* (**D**) mutants were imaged using transmission electron microscopy. Scale bars are 600 nm in the image and 200 nm in the inset.

One caveat to these assays is that they rely on immunoreactivity to specific linkages in the capsule. To address this limitation and to further confirm the loss of capsule, the presence of capsule on the cell surface was investigated using electron microscopy. In accordance with the previous results, no surface associated capsule was observed in the TIGR4 *spxB* mutant but was readily detectable on the surface of both the *lctO* mutant and the double mutant in patterns similar to the parental strain (Fig 5).

From these results, we conclude that deletion of *spxB* results in a defect in capsule biosynthesis in the TIGR4 background but not in the D39 background. These observations support our previous interpretation and suggest that the resistance to LL-37 of the *spxB* mutant in TIGR4 is primarily due to loss of capsule production. It is unknown if the absence of hydrogen peroxide plays an additional role in antimicrobial resistance. The TIGR4 double mutant, which produces minimal hydrogen peroxide, has some resistance to LL-37 although it has similar amount of capsule on its surface as the wild type. Moreover, in previous findings, the *spxB* mutant in an acapsular D39 R6 strain demonstrated slight resistance to antimicrobial killing [16]. Together, these data suggests that capsule plays the primary role and hydrogen peroxide may play a secondary role in sensitivity to antimicrobials.

### Confirmation of *spxB* Mutation

One potential mechanism for the lack of capsule in the TIGR4 background would be missense mutations in the capsule locus that results in altered capsule production or decreased abundance of the capsule transcripts. The restoration of the *spxB* mutant phenotype via complementation of *lctO* into the double mutant suggests that the loss of capsule was not due to secondary chromosomal mutations (S2 Fig). To further confirm this, we undertook whole genome sequencing and SNP analysis of the *spxB* mutant. No mutations in the capsule locus were detected compared to the reference TIGR4 genome (S1 File). In addition, we performed qRT-PCR for a number of genes in the capsule locus to include all operons [28]. No significant decrease in capsule transcript was detected among the strains (S1 Table), indicating that the lack of capsule was not due to transcriptional down-regulation. From these data, we conclude that the reduced capsule in the TIGR4 *spxB* mutant is not due to mutation of the capsule locus or loss of transcript abundance.

### Delineation of the Role of Hydrogen Peroxide in Virulence

**Fig 6:**
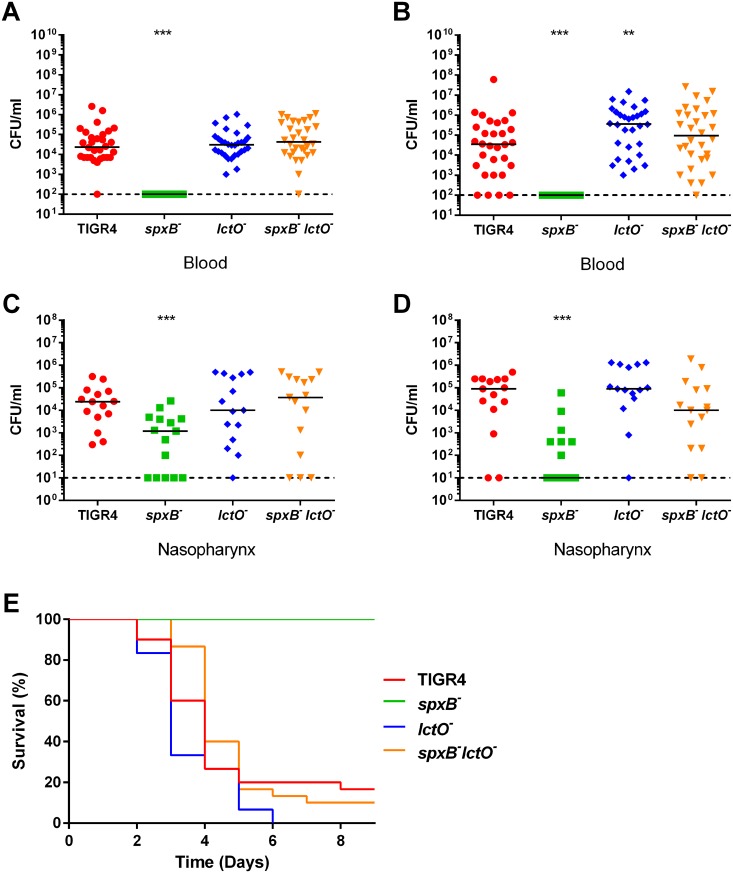
TIGR4 *spxB* mutant has reduced virulence in an IN mouse model. BALB/c mice were infected IN with 1 x 10^7^ cells. Bacterial presence in the blood in mice infected with TIGR4 strains was determined by detection of colony forming units (CFU/mL) at 24 hours (**A**) and 48 hours (**B**) post infection. In the same mice, bacterial carriage in the nasopharynx was determined at 24 hours (**C**) and 48 hours (**D**) post infection. Survival of mice was followed for 9 days (**E**). For each time-point of bacterial titers, mutant strains were compared to wild type using nonparametric Mann-Whitney t test; * p = 0.05–0.01, ** p = 0.01–0.001, *** p<0.001. Survival data were analyzed using the Mantel-Cox log rank test. p<0.0001 for TIGR4 *spxB* mutant compared to wild type; p = 0.0458 for TIGR4 *lctO* mutant compared to wild type; TIGR4 *spxB lctO* double mutant compared to wild type was non-significant.

The proposed role of hydrogen peroxide in pneumococcal pathogenesis has shown considerable variability, with mutation of *spxB* being attenuated in one serotype background while demonstrating heightened invasive potential in other serotypes [17, 19, 20, 29]. As previous studies have demonstrated that deletion of capsule renders the pneumococcus attenuated during invasive infection [30], we hypothesized that varying capsule biosynthesis in the *spxB* mutant in different serotypes could partially explain this inconsistency. To test this hypothesis, we investigated the virulence of the mutants in the serotype 4 and serotype 2 backgrounds in an intranasal murine model of infection. In the TIGR4 background, the *spxB* mutant was significantly attenuated as measured by both bacterial burden in the bloodstream (Fig 6A and 6B) and mouse survival (Fig 6E). Deletion of *lctO in the* TIGR4 background resulted in a slight, but significant increase in bacterial blood titers at 48 hours post challenge that resulted in a modest decrease in survival time compared to the parental TIGR4 (Fig 6B and 6E). The double mutant, which produces minimal hydrogen peroxide, was equivalent to the parental TIGR4 in terms of invasion into the bloodstream and resulted in similar mouse survival. Complementation of the double mutant with *lctO* expressed from a plasmid mimicked the *spxB* mutant phenotype in terms of bacterial burden in the blood and mouse survival (S2 Fig).

**Fig 7:**
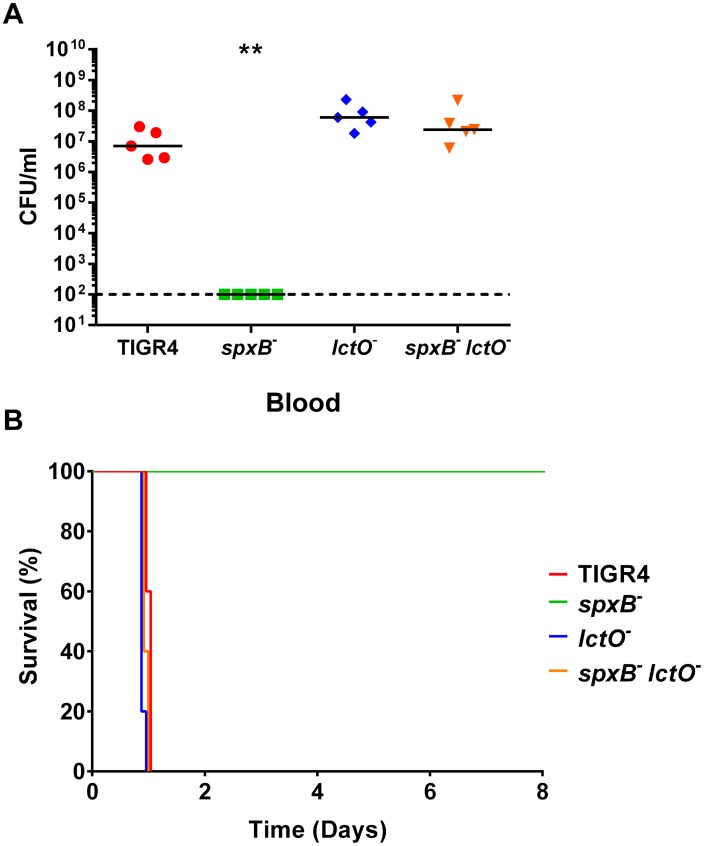
TIGR4 *spxB* mutant is not lethal in an IP mouse model. BALB/c mice were infected IP with 1 x 10^3^ cells and monitored for disease progression. Mice were infected with TIGR4 and the *spxB*, *lctO*, and double *spxB-lctO* mutants. Bacterial presence in the blood was determined at 24 hours (**A**) post infection. Survival of mice was followed for 8 days (**B**). For clarity purposes, the survival curves of TIGR4 and the *lctO* mutant was nudged. For blood titers, mutant strains were compared to wild type using nonparametric Mann-Whitney t test; ** p = 0.01–0.001. Survival data were analyzed using the Mantel-Cox log rank test. p = 0.0031 for TIGR4 *spxB* mutant compared to wild type; all other mutants compared to the wild type were non-significant.

Colonization of the nasopharynx is a prerequisite of streptococcal disease [31]. Reduced infectivity in an intranasal model of infection could result from reduced bacterial fitness in either the bloodstream or at the mucosal surface as the pneumococci typically colonize the nasal passages and subsequently translocate into the bloodstream resulting in bacteremia. To determine if the lower bacterial burden in the nasal passages (Fig 6C and 6D) was responsible for the observed reduction in virulence, we used an intraperitoneal (IP) murine model of infection, which provides the bacteria with more direct access to the bloodstream. In this model, the *spxB* mutant was significantly attenuated while the *lctO* mutant and double mutant maintained virulence in comparison to the TIGR4 parental strain in both bloodstream burden and survival (Fig 7). This result corroborates with the previous data and suggests that deletion of *spxB* negatively impacts dissemination of TIGR4 in the bloodstream. Because the double mutant is virulent, the observed reduction in virulence in the *spxB* mutant is likely due from the loss of the capsule and not from loss of hydrogen peroxide production.

**Fig 8:**
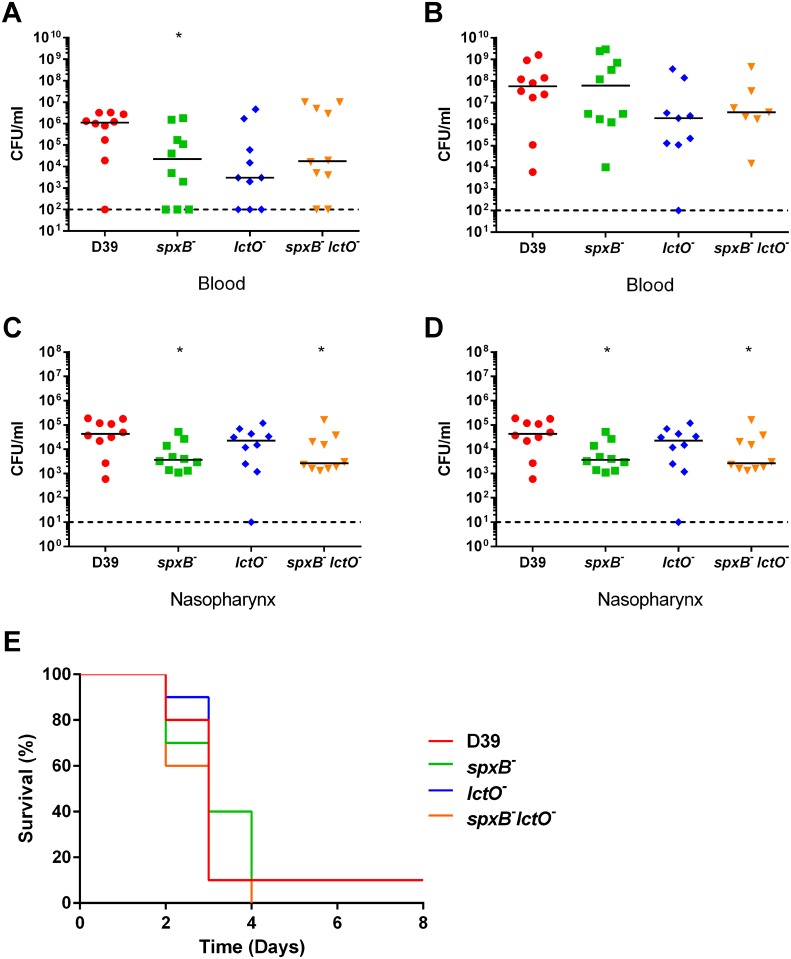
D39 *spxB* mutant maintains virulence in an IN mouse model. BALB/c mice were infected IN with 1 x 10^7^ cells. Bacterial presence in the blood in mice infected with D39 strains was determined at 24 hours (**A**) and 48 hours (**B**) post infection. In the same mice, bacterial carriage in the nasopharynx was determined at 24 hours (**C**) and 48 hours (**D**) post infection. Survival of mice was followed for 8 days (**E**). For each time-point of bacterial titers, mutant strains were compared to wild type using nonparametric Mann-Whitney t test; * p = 0.05–0.01. Survival data were analyzed using the Mantel-Cox log rank test. All mutants compared to the wild type were non-significant.

In the context of the D39 background, the three mutants had slightly reduced invasion in the bloodstream at 24 hours and nasopharynx carriage (Fig 8A, 8C and 8D). Yet, there was no significant difference in bloodstream burden at 48 hours (Fig 8B). The median blood titer values for D39 and the *spxB* mutant (S3 Table) is similar to that previously observed [29]. No detectable difference in mouse survival was observed for any of the mutants compared to the parental D39 (Fig 8E). When higher doses (1x10^8^) of D39 and the *spxB* mutant were used in the murine model, a slight but significant delay in mouse survival in those mice infected with the *spxB* mutant was observed, in concordance with previously reported results [19]. These data indicate that attenuation of the *spxB* mutant is strain dependent and correlates with capsule production, loss of which leads to resistance to the antimicrobial peptide LL-37.

### Effect of SpxB loss on Acetyl-CoA Levels

**Fig 9:**
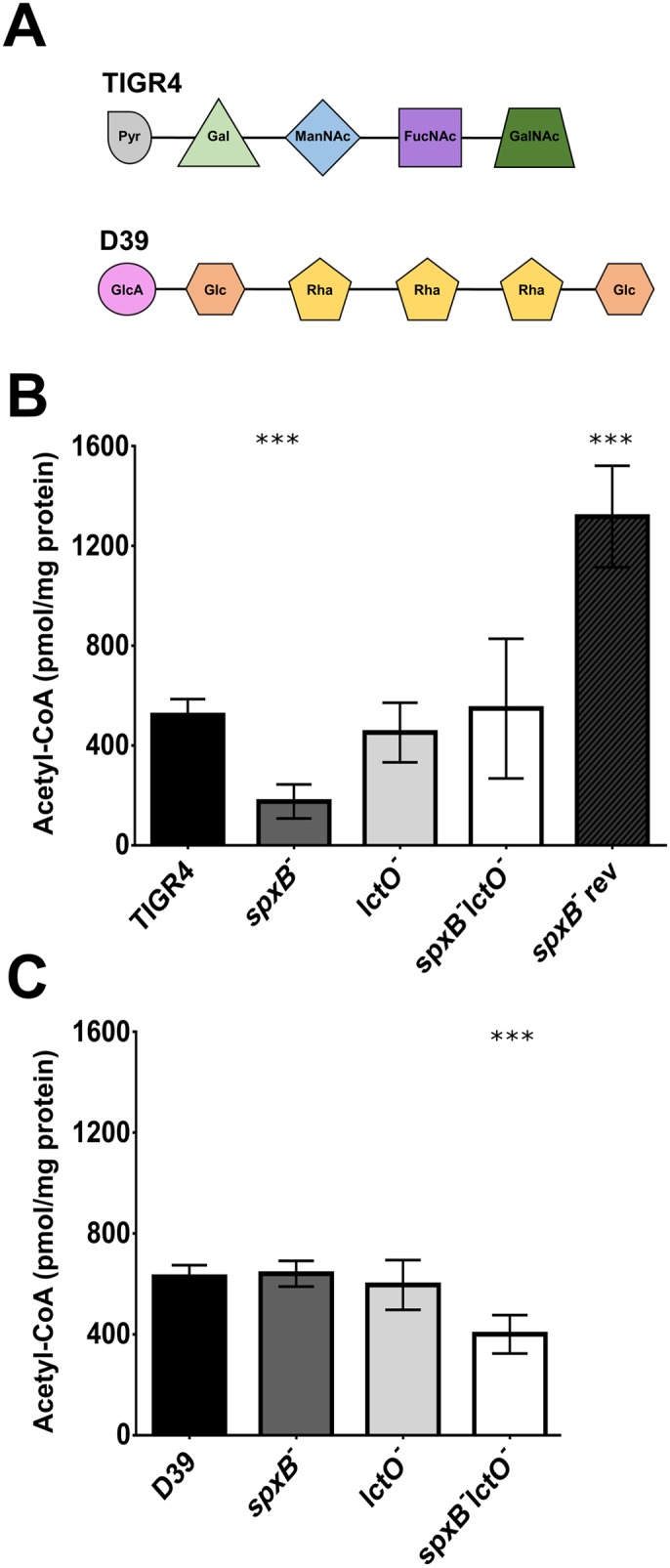
Acetyl-CoA steady-state levels were reduced in the TIGR4 *spxB* mutant. TIGR4 capsule consists of several sugars that are acetylated, unlike the capsule of D39. Capsule units are abbreviated: Pyr (pyruvate); Gal (galactose); ManNAc (N-acetyl-mannosamine); FucNAc (N-acetyl-fucosamine); GalNAc (N-acetyl-galactosamine); GlcA (glucuronic acid); Glc (glucose); Rha (rhamnose) (**A**). Acetyl-CoA levels were measured using mass spectrometry in wild type and the *spxB*, *lctO*, and double *spxB-lctO* mutants in the TIGR4 (**B**) and D39 (**C**) backgrounds. Acetyl-CoA values were normalized to total cellular protein and then plotted as pmol/ mg protein. Mutants were compared to the wild type using unpaired parametric t test; *** p<0.001.

One explanation for the disparity of capsule production in the *spxB* mutant between TIGR4 and D39 is the distinctly different types of carbohydrates used in the biosynthesis of the two serotypes [28]. TIGR4 incorporates several acetylated sugars into its capsule, while D39 uses none (Fig 9A). This is supported by the previous observation that deletion of *spxB* did not affect capsule production when the serotype 2 capsule was being produced in the TIGR4 genetic background (Fig 3). We hypothesized that, in the *spxB* mutant, a dysregulation of central metabolism leading to a reduction in the glycosyl acetyl-group donor, acetyl-CoA, has a negative impact on TIGR4 capsule biosynthesis [32–34]. To test this, we measured the intracellular levels of acetyl-CoA via mass spectrometry. The TIGR4 *spxB* mutant has greatly reduced levels (33%) of acetyl-CoA compared to the wild type (Fig 9B). The double mutant has levels of acetyl-CoA restored to wild type level and similar to the *lctO* mutant. In the D39 background, acetyl-CoA levels were similar between wild type and the *spxB* mutant (Fig 9C). Interestingly, the double mutant has about 36% lower acetyl-CoA levels than the wild type. These results indicate that deletion of *spxB* in the TIGR4, but not the D39, background alters metabolism resulting in reduced steady-state levels of acetyl-CoA, which is a critical co-factor necessary for type 4 capsule biosynthesis.

**Fig 10:**
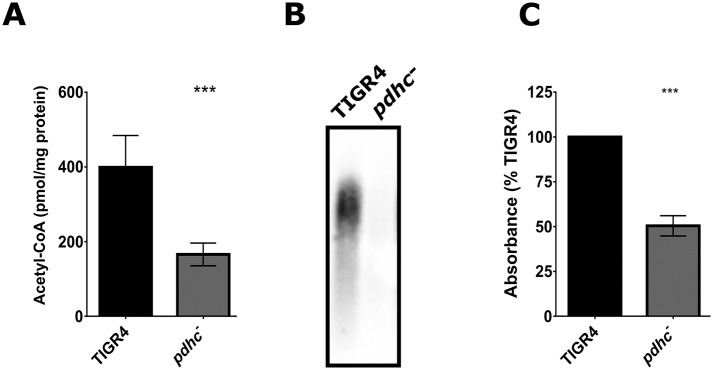
Disruption of *pdhc* reduces acetyl-CoA steady-state levels and abrogates capsule biosynthesis in TIGR4. Acetyl-CoA levels were measured in the wild type and the *pdhc* mutant in TIGR4 background (**A**). Measurement of capsule polysaccharide produced by the *pdhc* mutant was determined by capsule blotting (**B**) and whole-cell bacterial ELISA (**C**). Acetyl-CoA values were normalized to total cellular protein and then plotted as pmol/ mg protein. The mutant was compared to the wild type using unpaired parametric t test; *** p< 0.001.

To further substantiate this hypothesis, a deletion of a component of the pyruvate dehydrogenase complex (*pdhc*) was undertaken. This enzyme complex is responsible for converting pyruvate into acetyl-CoA via pyruvate decarboxylation and hence represents a secondary metabolic pathway for the generation of acetyl-CoA independent of pyruvate oxidase (SpxB) activity. Deletion of the pyruvate dehydrogenase complex resulted in both a significantly reduced level of acetyl-CoA (Fig 10A) as well as a defect in capsule biosynthesis (Fig 10B and 10C) that mirrored that in the *spxB* mutant. No loss of hydrogen peroxide production was observed in the *pdhc* mutant (S1 Fig). These data suggest that central metabolism, as evidenced by reduced intracellular acetyl-CoA levels, is a critical determinant for capsule biosynthesis in the serotype 4 TIGR4 background.

### Compensation for *spxB* Mutation

**Fig 11:**
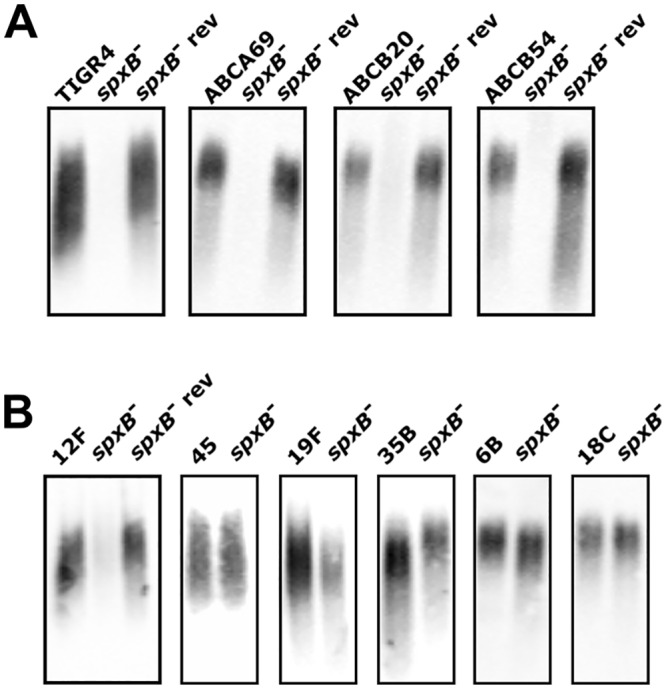
Loss of capsule in *spxB* mutant is conserved and secondary mutations can reverse this phenotype. Capsule production was measured using capsule blotting method. Strains used include wild type, *spxB* mutant, and *spxB* mutant revertant (rev) from four strains with serotype four capsule, including TIGR4, ABCA69, ABCB20, and ABCB54 (**A**). Capsule production was determined for *spxB* deletion in strains with capsules that contained different number of acetylated sugars, including 12F and the revertant and 45 (3 acetylated sugars), 19F and 35B (1 acetylated sugar), and 6B and 18C (0 acetylated sugars) (**B**).

SpxB and LctO play key roles in glucose metabolism (Fig 1) [23, 24]. We have shown here that deletion of *spxB* in TIGR4 alters metabolism and results in reduced acetyl-CoA levels, which may be expected to put considerable stress on the cell. To compensate for this dysregulation, it is possible that secondary suppressor mutations will occur. Indeed, disruption of acetate kinase, which converts acetate to acetyl-phosphate as an additional pathway to generate acetyl-phosphate independent of *spxB*, results in secondary mutations in *spxB*, in the positive regulator of *spxB (spxR)*, and in capsule biosynthesis genes [23]. Moreover, disruption of phosphoglucomutase, which converts glucose-6-P to glucose-1-P, leads to loss of capsule and a strong tendency for propagation of secondary mutations [35]. To determine if secondary mutations occur after deletion of *spxB* in TIGR4, we deleted *spxB* and identified spontaneous mutants that regained capsule production. Upon *spxB* deletion, we found a mixed population of two distinct phenotypes: small, dense, acapsular colonies and large, mucoid, capsular colonies. We then sequenced several colonies from each of these groups and identified any secondary mutations (S2 File). Of note, we found secondary mutations in a peptidase directly upstream of the pantetheine phosphate adenylyltransferase (stop), in the pyruvate dehydrogenase complex (missense), and in a serine/threonine kinase (missense). The *spxB* mutant with a secondary mutation in the peptidase, named *spxB* revertant, had a high level of acetyl-CoA at 2.5x the wild type (Fig 9B) and restored capsule synthesis (Fig 11A). These data suggest that there exists strong selective pressure for the development of compensatory mutations to alleviate the alterations in metabolism induced by deletion of *spxB*.

### Contribution of SpxB to Capsule Production amongst Diverse Serotypes

**Fig 12:**
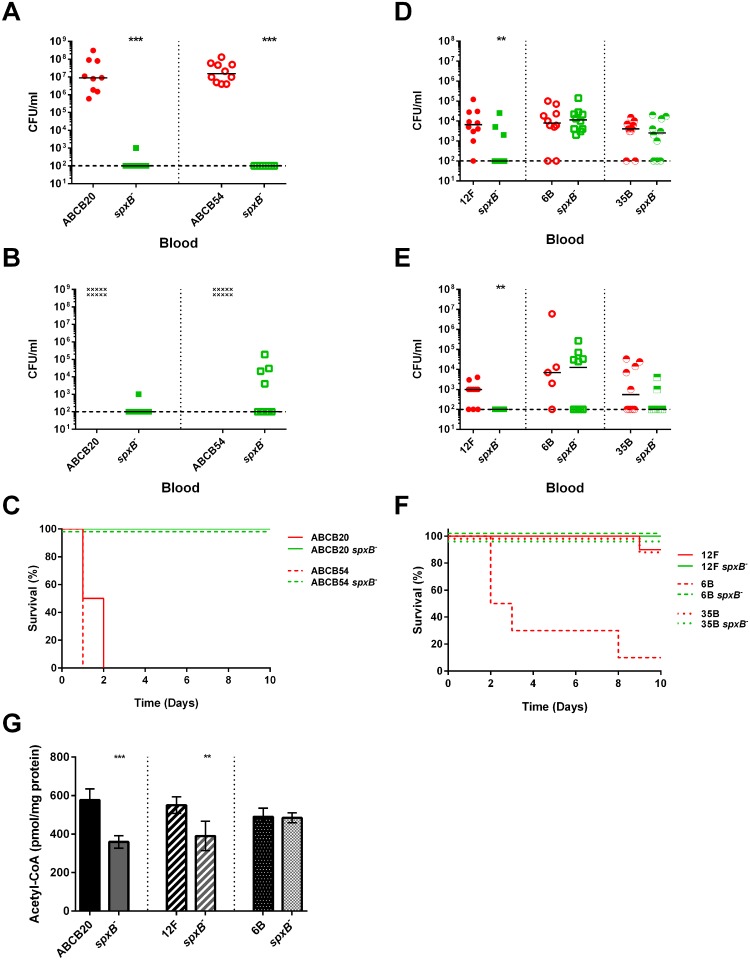
Deletion of *spxB* attenuates virulence and reduces steady-state acetyl-CoA in other pneumococcal strains with acetylated capsules. BALB/c mice were infected IP with 5 x 10^4^ cells. Bacterial presence in the blood in mice in strains with type 4 capsule was determined at 24 hours (**A**) and 48 hours (**B**) post infection and survival of mice was followed for 10 days (**C**). For strains with other capsule type—12F, 35B, 6B—bacterial presence in the blood in mice was determined at 24 hours (**D**) and 48 hours (**E**) post infection and survival of mice was followed for 10 days (**F**). Mice that did not survive to the 48 hour blood titer count are denoted by the symbol (x). Acetyl-CoA levels were measured in the wild type and *spxB* mutant in ABCB20, 12F, and 6B backgrounds (**G**). For each time-point of bacterial titers, mutant strains were compared to wild type using nonparametric Mann-Whitney t test; ** p = 0.01–0.001, *** p< 0.001. Survival data were analyzed using the Mantel-Cox log rank test. p< 0.0001 for ABCB20, ABCB54, and 6B *spxB* mutants compared to wild type; 12F and 35B *spxB* mutants compared to the wild type were non-significant. Acetyl-CoA values were normalized to total cellular protein and then plotted as pmol/ mg protein. The mutant was compared to the wild type using unpaired parametric t test; ** p = 0.01–0.001, *** p< 0.001.

The pneumococcus is an extremely genetically diverse pathogen in terms of both genomic content as well as specific loci, such as the capsule locus. With over 90 pneumococcal serotypes currently described, the encoding locus and the biochemical composition of the capsule reflects this diversity [28]. To determine if the relationship between acetylated capsules and deletion of *spxB* is conserved, we selected a subset of strains with capsule linkages identical to TIGR4 (other serotype 4 strains), strains with capsule types with similar acetylated linkages (serotypes 12F and 45), and additional serotypes distinct from serotype 4 in terms of composition and linkage groups (serotypes 6B, 18C, 19F, and 35B). Deletion of *spxB* resulted in undetectable capsule production in the strains with type 4 serotype (Fig 11A) and 12F serotype (Fig 11B). The revertant phenotype was observed in these strains as well. Surprisingly, the mixed population found in strains with type 4 and 12F serotypes was not observed in the *spxB* mutant in type 45 serotype; only the encapsulated *spxB* mutant was found (Fig 11B). It is possible that this strain has an inherent mechanism to compensate for the altered metabolism induced by loss of *spxB*, or that the stress induced by the *spxB* deletion makes the cells unstable and leads to rapid secondary mutations. In strains that have one acetylated sugar in their capsules (19F and 35B), the *spxB* mutants displayed detectable capsule but the absolute levels were reduced (Fig 11B). No discernable effect on capsule production was observed in strains with no acetylated sugars in its capsule (6B and 18C).

To test whether the defect of capsule in these other strains results in reduced virulence, we infected mice intraperitoneally with wild type and *spxB* mutant of two strains with type 4 capsules (ABCB20, ABCB54) and with type 12F, 6B, 35B, and 19F and monitored bacterial burden in the blood and mouse survival. For the strains with type 4 capsules, we observed a similar trend as TIGR4 where the *spxB* mutant had greatly diminished bacterial burden in the blood compared to the wild type (Fig 12A and 12B) and all of the mice infected with the *spxB* mutant in these backgrounds survived up to 10 days (Fig 12C). Mice infected with the 12F and 35B *spxB* mutant had lower bacterial burden in the blood compared to the wild type and survived to 10 days, while one of ten mice infected with wild type did not survive (Fig 12D–12F). Similar to D39 infection, mice infected with the *spxB* mutant in 6B, which has no acetylated sugars in the capsule, had similar blood titers as those infected with wild type (Fig 12E). Surprisingly, although the bacterial burden is similar at early time points, mice infected with the *spxB* mutant survived while those infected with wild type did not. No bacterial burden or mortality was observed for mice infected with 19F wild type or *spxB* mutant. These data indicate that while there is a clear link between the relationship of *spxB* and capsule production to virulence, it is likely that *spxB* has other contributions to pathogenesis in a strain dependent manner.

Previous results suggest that the *spxB* deletion results in altered central metabolism as indicated by reduced acetyl-CoA levels in strains with acetylated capsules (Fig 9). To test this hypothesis, we measured acetyl-CoA levels of strains with capsule types 4 (ABCB20), 12F, and 6B which have capsules with three, three, and zero acetylated sugars, respectively (Fig 12G). The *spxB* mutant in both ABCB20 and 12F had significantly reduced levels of acetyl-CoA compared to wild type, resembling that observed in the *spxB* mutant in TIGR4. Loss of *spxB* in 6B did not affect acetyl-CoA levels, in concordance with our observations in D39. Taken together, these data support our hypothesis of a relationship between SpxB function and utilization of acetylated sugars in capsule biosynthesis.

## Discussion

A striking difference between strains of pneumococci is the chemical diversity of the polysaccharide capsule. The capsule is one of the most important virulence factors from both a pathogenesis and a therapeutic standpoint as it is the target for the only commercially available vaccines against invasive pneumococcal disease [36]. This highly immunogenic surface structure shows striking variability between strains in both the components incorporated and the linkages between the sugars comprising the basic structure. This variability underscores the capacity of the pneumococcus to rapidly evade capsule based vaccines through both serotype replacement and capsular switching through recombination events among highly conserved genes flanking the capsule locus [37, 38].

As a means of immune evasion, the capsule is a necessary expenditure of energy for the pneumococcus within the human host, as non-encapsulated strains are typically rapidly cleared—though there are certain exceptions for particular tissue tropisms such as isolates from conjunctivitis cases [39, 40]. Moreover, there is an intricate relationship between capsule type and the metabolic cost associated with that type. It is not surprising, then, that certain capsular exchanges can cause corresponding fitness defects, both *in vitro* and during nasopharyngeal carriage impacted by both the number of carbons per polysaccharide repeat as well the charge of the capsule [41]. The tradeoff of sustaining growth for incorporating specific carbohydrates into the capsule is reflected by replication defects in nutrient poor media for those strains with more energetically expensive capsule types (in terms of the number of carbons per repeat unit of polysaccharide) and is hypothesized to reflect capsule type prevalence during carriage [21]. The diversity of metabolite requirements may also play a role in the simultaneous carriage of multiple serotypes, which is becoming increasingly appreciated as culture-independent detection methods are employed [42]. Hence, the pneumococcal capsule is under considerable selective pressure, both from a metabolic perspective in terms of incorporation of carbohydrates that could otherwise support energy production and replication as well as from the host immune system during both carriage and vaccination.

It is becoming increasingly evident that bacterial metabolic networks and virulence share an intimate relationship. Sensing of nutrient availability can drive the regulation and production of virulence factors in a multitude of bacterial pathogens. The capsule represents an energetically intensive structure to synthesize as carbohydrates incorporated into the backbone could otherwise be shunted to glycolysis to support replication [21]. Hence, it is not surprising that capsule biosynthesis is integrated into the metabolic regulatory networks of the pneumococcus [43, 44]. The metabolic capacity of pneumococcus, like other streptococci, is primarily limited to glycolysis due to a lack a complete TCA cycle as well as an electron transport chain. The activity of pyruvate oxidase has a direct impact on ATP synthesis and carbon utilization through the glycolysis pathway in that it converts pyruvate to acetyl-phosphate, generating a molecule of hydrogen peroxide from oxygen in the process (Fig 1). The acetyl-phosphate then can be converted to acetate via acetate kinase or to acetyl-CoA via phosphate acetyltransferase. Loss of pyruvate oxidase has several downstream effects, including hydrogen peroxide production, and fatty acid metabolism [45]. Here, we show that loss of pyruvate oxidase impacts capsule production in a serotype specific manner. Of note, previous findings suggest that deletion of *spxB* in D39 leads to about 20% increase in capsule production [18]. This finding differs from what is observed here where no discernible increase was observed. One possible explanation is method of detection as in the previous studies, glucuronic acid was used as a measurement of capsule production. While glucuronic acid is incorporated into the capsule, it is also used in other key metabolic processes and observed differences could potentially be confounded by other metabolites. Of note is the observation of an increase in serotype 2 capsule production in the capsule-swapped strains when *spxB* is deleted, in agreement with previous observations. Previous reports on loss of *spxB* have suggested that lowered acetyl-phosphate and ATP levels could be responsible for the downstream effects either by having a direct impact [22] or indirectly by affecting two-component systems [46, 47]. However, in this investigation, we also analyzed acetyl-phosphate and ATP levels in TIGR4 (S3 Fig). Lower levels were observed in both the *spxB* mutant and the double mutant. As the double mutant retains wild type levels of acetyl-CoA, capsule production, and virulence, the loss of capsule in the *spxB* mutant did not correlate with absolute levels of acetyl-phosphate or ATP.

Alternatively, the reduction of acetyl-CoA upon loss of SpxB could lead to downstream effects on fatty acid metabolism and capsule biosynthesis in that acetyl-CoA plays an important role in both of these pathways. In this study, we find that steady state acetyl-CoA levels are reduced upon loss of SpxB only in those strains with acetylated sugars incorporated into the capsules (type 4 and 12F). Strains without acetylated sugars incorporated into their capsules (type 2 and 6B) may have altered metabolic pathways that, without the drain on acetyl-CoA for capsule synthesis, the acetyl-CoA cellular flux is lower and that these strains more readily relieve the stress from loss of SpxB to maintain the acetyl-CoA pool. One interpretation of the lower acetyl-CoA levels observed in strains with acetylated sugars in their capsule is that the absence of SpxB or PDHC limits the conversion of glucose to acetyl-CoA. This lower steady-state of acetyl-CoA may have a direct impact on the formation of the acetylated sugars required for capsule production or it may reflect lower overall flux through glycolysis. The data in this study does not allow a definitive description of how the overall metabolic network is impacted by the loss of *spxB*. Indeed, from this data it is unclear why loss of *lctO* in the *spxB* mutant restores acetyl-CoA levels and capsule synthesis. One possible explanation is lactate levels build up due to the loss of lactate oxidase. This excess lactate could lead to a feedback inhibition that inhibits lactate dehydrogenase activity, thereby increasing readily available pyruvate which can be converted to acetyl-CoA via the pyruvate dehydrogenase complex. However, from these data, it is clear that the requirement of *spxB* for virulence is not due to reduced hydrogen peroxide production in that 1) the double mutant produced minimal hydrogen peroxide but is virulent and 2) the *pdhc* mutant produces wild type levels of hydrogen peroxide but has a loss of capsule similar to the *spxB* mutant.

Multiple human pathogens encode an extracellular capsule and in many cases the virulence of the pathogen is associated with the expression of this structure. Similar links between metabolism and capsule production are observed in other pathogens, such as *Streptococcus pyogenes* whereby zinc stress perturbs both central metabolism as well as capsule biosynthesis [48]. The most prevalent strains of *Staphylococcus aureus* have capsule with acetylated sugars similar to TIGR4 (FucNAc and ManNAcA, which is generated by oxidation of ManNAc) [49]. *S*. *aureus* has a pyruvate oxidase that affects acetate metabolism [50]. However, the pyruvate oxidase converts pyruvate to acetate directly, unlike in *S*. *pneumoniae*, and the effect on acetyl-CoA and capsule production is unknown. Group B streptococci also acetylates sialic acid, which is then incorporated into their capsule, a modification that is also required for virulence [51, 52]. However, unlike *S*. *pneumoniae*, group B streptococci lack pyruvate oxidase and hence this pathway is inoperative. Group B streptococci can generate acetyl-CoA through the activities of pyruvate dehydrogenase complex. *Neisseria meningitidis* virulence requires production of capsule and several serotypes have acetylated capsules which also rely on production of acetyl-CoA [53, 54]. *N*. *meningitidis* also lacks pyruvate oxidase and can also generate acetyl-CoA from pyruvate via the pyruvate dehydrogenase complex. We have shown here that deletion of the pyruvate dehydrogenase complex also abrogates capsule production in *S*. *pneumoniae* TIGR4. As such, deletion of such enzymatic pathways in Group B streptococci and *N*. *meningitidis* may also impair capsule biosynthesis in a manner similar to what is observed in pneumococci with acetylated capsules. The capsule of *Cryptococcus neoformans* also contains acetylated sugars and a loss of a key metabolic enzyme, ATP-citrate lyase, which converts citrate to acetyl-CoA, results in loss of capsule production and reduced virulence, similar to what we observe here in *S*. *pneumoniae* [55]. These data underscore the intricate balance between metabolite bioavailability and the biosynthesis of the complex cellular structures required for bacterial survival within the human host.

## Materials and Methods

### Media and growth conditions

*Streptococcus pneumoniae* was grown on tryptic soy agar (EMD Chemicals, New Jersey) supplemented with 3% sheep blood or in C+Y, a defined semi-synthetic casein liquid media [56] supplemented with 0.5% yeast extract. Cultures of *S*. *pneumoniae* were inoculated from either frozen stock or newly streaked TSA blood pates and incubated at 37°C in 5% CO_2_. Strains used in this study are listed in S4 Table.

### Bacterial Constructs

The *spxB* (Sp_0730), *lctO* (Sp_0715), and *pdhc* (E1 alpha and beta subunits; Sp_1163 and Sp_1164) mutants were created via splicing by overhang extension PCR (SOE-PCR) [57]. Briefly, 1-kb upstream and downstream fragments of the target gene were amplified and spliced to either an erythromycin (*spxB*) or spectinomycin (*lctO*, *pdhc*) cassette amplified using primers Erm_F/ Erm_R and Spec_F/ Spec_R, respectively. SOE-PCR products were subsequently transformed into the TIGR4 and D39 strains of *S*. *pneumoniae*. Knockout mutants selected by antibiotic resistance to erythromycin (1 μg/mL) or spectinomycin (150 μg/mL) were verified by PCR to confirm insertion of the SOE-PCR product and deletion of the target gene. The double *spxB lctO* mutant was generated by transforming the *lctO* SOE-PCR product into the *spxB* mutant background; mutants were selected by spectinomycin resistance and both gene deletions were verified by PCR. Primers used to make mutants are listed in S5 Table. The capsule swapped strain (TIGR4::D39) was generated by first replacing the capsule locus of TIGR4 with the SweetJanus cassette by transforming TIGR4 with genomic DNA of SpnYL001 [58] and selection on kanamycin (400 ug/mL), followed by transformation with D39 genomic DNA and selection on 10% sucrose. Deletion of *spxB* was performed as described above.

### Hydrogen Peroxide Production

Production of hydrogen peroxide was determined in two methods. In the first, hydrogen peroxide was measured by the Amplex Red kit (Thermo Scientific). Cells were grown to mid-logarithmic phase (OD_620_ = 0.4), pelleted, and resuspended in PBS. Cells were incubated for 20 minutes at room temperature to allow for hydrogen peroxide production and then pelleted. The hydrogen peroxide present in the supernatant was measured according the kit instructions in a 96 well plate; each strain was added in duplicate in each plate, which was repeated in triplicate. Values were normalized using total cell protein as determined by BCA assay (Pierce). Hydrogen peroxide production was compared using unpaired parametric t-test in Prism 6. In the second method, hydrogen peroxide production was observed by colorimetric changes of the cells on Todd Hewitt yeast plates plus horseradish peroxidase (0.2 mg/mL) and 2,2'-azino-bis(3-ethylbenzothiazoline-6-sulphonic acid) (3 mg/mL). Strains were grown to mid-logarithmic phase in C+Y, serially diluted, plated, and incubated in an anaerobic chamber overnight at 37°C. After exposure to ambient air, colonies that produce hydrogen peroxide turned purple.

### LL-37 Killing Assay

Strains were grown to mid-logarithmic phase (OD_620_ = 0.4) in C+Y and frozen at ^-^80°C. Cells were then thawed and back-diluted 1:60 in 300 μL C+Y plus LL-37 (diluted in C+Y to a final concentration of 0, 10, 21 and 42 μg/mL) in a 96-well plate. Growth at 37°C in 5% CO_2_ was monitored using Biotek Cytation 3 for 30 hours. Each strain was grown in duplicate per plate, which was repeated four times.

### Capsule Blotting

Capsule presence was detected using the capsule blotting method [27]. Briefly, strains were grown to mid-logarithmic phase (OD_620_ = 0.4) in C+Y and were lysed using DOC/SDS disruption of the membrane. Proteins were removed by incubation with proteinase K; a small sample of the cell lysate was used to measure protein concentration prior to protein degradation via BCA assay (Pierce). Cell lysates were diluted to 22.5 mg/mL cellular protein and subjected to agarose gel electrophoresis. The samples were transferred to a nitrocellulose membrane following standard capillary transfer in a high-salt buffer. The membrane was then probed with serotype-specific antibodies (Statens Serum Institute) and HRP conjugated secondary antibody (Bio-Rad), followed by detection using a HRP chemiluminescent substrate (Thermo Scientific).

### Bacterial ELISA

Whole-cell bacterial ELISA was performed as a quantitative measure of capsule on the bacterial surface. Cells were grown to mid-logarithmic phase (OD_620_ = 0.4) in C+Y, pelleted, and diluted 1:5 in coating buffer (sodium carbonate). The bacteria were bound to the plate via centrifugation. After the plate dried overnight at room temperature and was subjected to blocking buffer (10% FBS), the cells were probed with serotype-specific antibodies (Statens Serum Institute) and AP conjugated secondary antibody (Southern Biotech), followed by detection using an AP yellow ELISA substrate (Sigma). Each strain was repeated in duplicate in each plate, which was repeated three times. Immunoreactivity was compared using unpaired parametric t test in Prism 6.

### Fluorescence Microscopy

Capsule was further detected using fluorescence microscopy. Cells were grown to mid-logarithmic phase (OD_620_ = 0.4) in C+Y, pelleted, and fixed in 4% paraformaldehyde. Cells were then incubated with serotype-specific antibodies (Statens Serum Institute) in 5% FBS and 0.2% Saponin, followed by incubation with Alexa 488 secondary (Thermo-Fisher). Stained cells were mounted onto coverslips with Prolong Gold with DAPI (Thermo-Fisher) and imaged using a Nikon C2 confocal microscope at 60X resolution.

### Electron Microscopy

Cell surface structures were imaged using transmission electron microscopy. Strains were grown to mid-logarithmic phase (OD_620_ = 0.4) in C+Y and then promptly pelleted. The samples were fixed in 2.5% glutaraldehyde in 0.1M sodium cacodylate buffer, pH 7.4, and post fixed in 2% osmium tetroxide in 0.1M cacodylate buffer with 0.3% potassium ferrocyanide for 1.5 hours. After rinsing in the same buffer, the cells were dehydrated through a series of graded ethanol to propylene oxide, infiltrated and embedded in epoxy resin, and polymerized at 70°C overnight. Semi thin sections (0.5 micron) were stained with toluidine blue for light microscope examination. Ultrathin sections (80nm) were cut and imaged using an FEI Tecnai 200Kv FEG Electron Microscope with an ATM XR41 Digital Camera.

### qRT-PCR Analysis

RNA was extracted from cells grown to mid-logarithmic phase (OD_620_ = 0.4) using the QiaShredder and RNeasy Kit (Qiagen) by following the kit instructions with the exception of the lysis step, which was performed by membrane disruption using zirconia/silica beads. cDNA was synthesized from random hexamers using Superscript First Strand Synthesis kit (Invitrogen) as per the instructions. qRT-PCR was performed with SYBR green (Thermo Scientific) using primers for *spxB*, *lctO*, and four genes from the TIGR4 capsule locus that cover each operon and include *cps4A* (Sp_0346), *cps4E* (Sp_0350), *mnaA* (Sp_0357), and *fnlC* (Sp_0360). Strains were extracted in triplicate and qRT-PCR was repeated in triplicate for each extraction. CT values for each gene were normalized to that of *gyrA*. To test alterations in expression of oxidative response genes, RNA was extracted and qRT-PCR was performed as described above for *sodA* (Sp_0766), *tpxD* (Sp_1651), and *ertX1* (Sp_0659) after cells were grown to mid-logarithmic phase (OD_620_ = 0.4) followed by incubation with or without 16 ug/ml LL-37 for 30 minutes.

### Acetyl CoA Measurement

Intracellular acetyl-CoA levels were measured using mass spectrometry. Cells were grown to mid-logarithmic phase (OD_620_ = 0.4) in C+Y. Cells were pelleted at 6,000 x g for 5 min at room temperature and then lysed using methanol/ 2% acetic acid and subjected to chloroform treatment. 250 pmol of [^13^C_2_] acetyl-CoA (Sigma) was added. Metabolites were extracted from the aqueous phase via a 2-(2-pyridyl) ethyl column, eluted using 95% ethanol/ 50mM ammonium formate, and blown under nitrogen for analysis. Samples were resuspended in 90% methanol + 15mM ammonium hydroxide. Mass spectrometry of acetyl-CoA was performed using a Finnigan TSQ Quantum (Thermo Electron) triple-quadrupole mass spectrometer. The instrument was operated in positive mode using single ion monitoring (SIM) neutral loss scanning corresponding to the loss of the phosphoadenosine diphosphate from CoA species. The ion source parameters were as follows: spray voltage, 4,000 V; capillary temperature, 250°C; capillary offset, −35 V; sheath gas pressure, 10; auxiliary gas pressure, 5; tube lens offset was set by infusion of the polytyrosine tuning and calibration in electrospray mode. Acquisition parameters were as follows: scan time, 0.5 s; collision energy, 30 V; peak width Q1 and Q3, 0.7 FWHM; Q2 CID gas, 0.5 mTorr; source CID, 10 V; neutral loss, 507.0 m/z; SIM mass of 810 m/z with a scan width of 8 m/z to capture the signal from light and heavy acetyl-CoA. Acetyl-CoA levels were compared using unpaired parametric t test in Prism 6.

### ATP and Acetyl-Phosphate Measurement

Cells were grown in 40 mL C+Y to OD_620_ = 0.3 and then lysed via mechanical disruption of the membrane using glass beads in a Fast Prep (MP Biomedicals). Total protein concentration was measured using BCA assay (Pierce). Cell debris was removed by filtration using Amicon 10K filters (EMD Millipore). Intracellular ATP levels were measured from the purified lysates using Cell Titer Glo Assays (Promega). Acetyl-phosphate in the lysates was converted to ATP as previously described [23] and quantitated using Cell Titer Glo Assays. This protocol was repeated four times and each sample was plated in duplicate. ATP and acetyl-phosphate levels were compared using unpaired parametric t test in Prism 6.

### Confirmation of *spxB* mutation and secondary mutations

To confirm that a secondary mutation in the capsule locus did not occur upon mutation of *spxB*, we extracted the genomic DNA from the *spxB* mutant and performed whole genome sequencing on the Illumina HiSeq. Libraries were prepared using Nextera library preparation kits and sequenced according to manufacturer’s specification. A total of ~13 million pairs of reads (101 bp x 2) was obtained for each sample from Illumina sequencing. Read quality was assessed by FASTQC. Low-quality bases and/or reads were trimmed using Trimgalore. Around 2% of the reads in each sample was excluded from downstream analysis due to low read quality. The high quality reads were aligned against the reference genome (GenBank ID: GCF_000273455.1) using the MEM algorithm of BWA with default setting [51]. The alignment coverage depth is ~1000x on average. SNPs were identified by SAMtools and called subsequently by BCFtools [59]. The threshold of coverage was set as minimum of 50. The SNPs with an associated PHRED quality score of ≥50 were considered robust. No mutation was detected in the capsule synthesis operon (GCF_000273455.1: 320,532–341,536).

To confirm the phenotype of the *spxB* mutant is reproducible, we repeated the *spxB* SOE-PCR transformation. Upon mutation, TIGR4 demonstrated two phenotypes: small, dense colonies and large, mucoid colonies. The colonies were grown in C+Y and tested for capsule synthesis by reactivity via quelling reaction with latex serotype-specific antisera (Statens Serum Institute). The small colonies had little reactivity with the antisera, while the large colonies had reactivity similar to the wild type. To identify genotypic differences between these two phenotypes, we extracted genomic DNA and performed whole genome sequencing and SNP analysis for over twenty of each colony type. The Illumina reads processing and SNP analysis followed the method as described previously. To see if this dual colony morphology was conserved, we mutated *spxB* in other serotypes—including ABCA69 (4), ABCB20 (4), ABCB54 (4), CDC007 (6B), CDC030 (12F), CDC048 (18C), ABCA31 (35B), and Sp128 (45)—in the same manner as described above.

### Mouse Studies

For bacterial burden and survival studies, strains were grown in C+Y media to an OD_620_ of 0.4 and diluted according to a previously determined standard curve. Bacteria were enumerated to assure that the proper amount of bacteria was used in infection. Bacteria were introduced into 7-week-old female BALB/c mice (Jackson Laboratory) via intranasal (IN) administration of 10^7^ CFU of bacteria in PBS (30 μL) or intraperitoneal (IP) administration of 1 x 10^3^ for TIGR4 strains or 5 x 10^4^ for clinical strains CFU of bacteria in PBS (100 μL). Mice were monitored for disease progression and euthanized via CO_2_ asphyxiation. Blood for titer determination was collected via tail snip at 24 and 48 hour post-infection and subsequent serial dilution and plating. Bacteria colonizing the nasopharynx were collected by insertion and removal of PBS (20 uL) into the nasal cavity. Survival data were analyzed using the Mantel-Cox log rank test in Prism 6. Bacterial titers were compared using nonparametric Mann-Whitney t test in Prism 6.

### Ethics Statement

All experiments involving animals were performed with prior approval of and in accordance with guidelines of the St. Jude Institutional Animal Care and Use Committee. The St Jude laboratory animal facilities have been fully accredited by the American Association for Accreditation of Laboratory Animal Care. Laboratory animals were maintained in accordance with the applicable portions of the Animal Welfare Act and the guidelines prescribed in the DHHS publication, Guide for the Care and Use of Laboratory Animals. All mice were maintained in BSL2 facilities and all experiments were done while the mice were under inhaled isoflurane (2.5%) anesthesia. Mice were monitored daily for signs of infection. This work was approved under the IACUC protocol number 538-100013-04/12 R1.

## Supporting Information

S1 Table qRT-PCR. Mean CT values with standard deviation of six genes.
(DOCX)

S2 Table qRT-PCR. Mean CT values with standard deviation of oxidative stress response genes with and without LL-37.
(DOCX)

S3 Table Median Log values of bacterial burden. (DOCX)

S4 Table Strains used in this study. (DOCX)

S5 Table Primers used in this study. (DOCX)

S1 File Whole genome sequencing and SNP analysis comparing the *spxB* mutant to wild type TIGR4. *spxB* mutant was sequenced twice and was compared to the TIGR4 genome in the NCBI database (tab 1) and to the TIGR4 strain used in these studies (tab 2). Identified SNPs are listed: *spxB* mutant (columns J and K).
(XLSX)

S2 File Whole genome sequencing and SNP analysis comparing multiple colonies of the *spxB* mutant to wild type TIGR4. Colonies of the two phenotypes of the *spxB* mutant and the TIGR4 strain used in these studies were sequenced and were compared to the TIGR4 genome in the NCBI database. Identified SNPs are listed: TIGR4 (columns K and L); twenty small acapsular *spxB* mutant colonies (columns M through AF); eighty large capsular *spxB* mutant colonies (AG through CN).
(XLSX)

S1 Fig Hydrogen peroxide production is reduced in the *spxB* mutant and is negligible in the double mutant in TIGR4 and D39. Hydrogen peroxide production was determined in two methods. Strains include wild type and the *spxB*, *lctO*, and the *spxB lctO* double mutant in the TIGR4 (**A**, **C, F**) and D39 (**B**, **D, F**) backgrounds, as well as the TIGR4 *lctO* mutant and double mutant complemented with pABG5-lctO (*lctO* ^*-/+*^ and *spxB* ^*-*^ *lctO* ^*-/+*^) (**A, C**), the TIGR4 *pdhc* mutant (**E**), and the wild type and *spxB* mutant in the TIGR4 with D39 capsule (**F**). Production by colonies on plates was indicated by purple coloration (**A**, **B**). Strains were serially diluted 1:10 from left to right. Production in cell culture was determined using the Amplex Red kit (**C**, **D, E, F**). Values were normalized to total cell protein and then plotted as percentage of wild type, where TIGR4 produces 1.87 ± 0.27 μM/μg cellular protein and D39 produces 2.11 ±1.08 μM/μg cellular protein. Hydrogen peroxide production was compared using unpaired parametric t test; all mutants compared to wild type were significant with p<0.001.
(DOCX)

S2 Fig Complementation of *lctO* to the double mutant restores the *spxB* mutant phenotype. Functional complementation with pABG5-lctO was determined. Strains include TIGR4 and the *spxB*, *lctO*, and *spxB lctO* mutants, and the *lctO* mutant and double mutant complemented with pABG5-lctO (*lctO* ^*-/+*^ and *spxB* ^*-*^ *lctO* ^*-/+*^). Capsule production was measured using capsule blotting method (**A**). BALB/c mice were infected IN with 1 x 10^7^ cells and monitored for disease progression (**B-F**). Survival of mice was followed for 8 days (**B**). For clarity purposes, the survival curves of the *spxB* ^*-*^ *lctO* ^*-/+*^ mutant was nudged. Bacterial presence in the blood was determined at 24 hours (**C**) and 48 hours (**D**) post infection. In the same mice, bacterial carriage in the nasopharynx was determined at 24 hours (**E**) and 48 hours (**F**) post infection. The titer and survival data for the wild type and mutants was used in conjunction with other mouse studies in Figs 4 and 5 and are included here for comparison with the results from mice infected with the complemented strains, which were performed at the same time. Survival data were analyzed using the Mantel-Cox log rank test. p = 0.0018 for *spxB* mutant and the complemented double mutant compared to wild type; p = 0.0394 for the double mutant compared to wild type; the *lctO* mutant and complement compared to the wild type were non-significant; the complemented double mutant compared to the *spxB* mutant was non-significant. For blood titers, mutant strains were compared to wild type using nonparametric Mann-Whitney t test; * p = 0.05–0.01, ** p = 0.01–0.001.
(DOCX)

S3 Fig ATP and acetyl-phosphate steady-state levels are reduced in the *spxB* and double mutants. Intracellular steady-state levels of ATP (**A**) and acetyl-phosphate (**B**) were determined. Strains include TIGR4 and the *spxB*, *lctO*, and *spxB lctO* mutants. Values were normalized to total cell protein and then plotted as percentage of wild type. ATP and acetyl-phosphate levels were compared using unpaired parametric t test; **, p = 0.01–0.001; ***, p<0.001.
(DOCX)
