# Supplementary material for: Pyruvate Oxidase as a Critical Link between Metabolism and Capsule Biosynthesis in Streptococcus pneumoniae
Source: PLoS Pathog. 2016 Oct 19;12(10):e1005951. doi: 10.1371/journal.ppat.1005951 (PMC5070856; doi:10.1371/journal.ppat.1005951)
Supplement: S1 Table — Mean CT values with standard deviation of six genes. (DOCX) [file ppat.1005951.s001.docx]

**S1 Table. qRT-PCR. Mean CT values with standard deviation of six genes.**

|  | **TIGR4** | **TIGR4 *spxB*^-^** | **TIGR4 *lctO*^-^** | **TIGR4 *spxB*^-^ *lctO*^-^** |
| --- | --- | --- | --- | --- |
| ***spxB*** | 12.40 (0.08) | 29.58 (1.69) | 12.23 (0.52) | 20.88 (0.34) |
| ***lctO*** | 14.31 (0.22) | 15.11 (0.14) | 35.04 (1.58) | 30.81 (0.36) |
| ***cps4A*** | 14.96 (0.33) | 13.92 (0.31) | 15.05 (0.19) | 14.41 (0.17) |
| ***cps4E*** | 14.58 (0.14) | 14.49 (0.06) | 14.60 (0.10) | 14.50 (0.15) |
| ***mnaA*** | 15.08 (0.32) | 14.85 (0.34) | 15.06 (0.48) | 15.19 (0.24) |
| ***fnlC*** | 16.78 (0.10) | 16.08 (0.10) | 16.79 (0.03) | 16.32 (0.22) |
|  |  |  |  |  |
|  |  |  |  |  |
