# Supplementary material for: Pyruvate Oxidase as a Critical Link between Metabolism and Capsule Biosynthesis in Streptococcus pneumoniae
Source: PLoS Pathog. 2016 Oct 19;12(10):e1005951. doi: 10.1371/journal.ppat.1005951 (PMC5070856; doi:10.1371/journal.ppat.1005951)
Supplement: S2 Table — Mean CT values with standard deviation of oxidative stress response genes with and without LL-37. (DOCX) [file ppat.1005951.s002.docx]

**S2 Table. qRT-PCR. Mean CT values with standard deviation of oxidative stress response genes with and without LL-37.**

|  | **TIGR4** | **TIGR4 *spxB*^-^** | **TIGR4 *lctO*^-^** | **TIGR4 *spxB*^-^ *lctO*^-^** |
| --- | --- | --- | --- | --- |
| **-LL37** |  |  |  |  |
| ***sodA*** | 15.64 (0.08) | 15.59 (0.27) | 16.51 (0.06) | 17.31 (0.10) |
| ***tpxD*** | 16.04 (0.10) | 16.85 (0.03) | 17.72 (0.06) | 19.55 (0.34) |
| ***ertX1*** | 21.76 (0.37) | 20.89 (0.27) | 18.92 (0.11) | 18.90 (0.29) |
| **+LL37** |  |  |  |  |
| ***sodA*** | 14.96 (0.04) | 15.75 (0.10) | 15.53 (0.29) | 16.69 (0.16) |
| ***tpxD*** | 15.75 (0.17) | 16.95 (0.43) | 17.29 (0.09) | 19.06 (0.21) |
| ***ertX1*** | 21.00 (0.08) | 21.61 (0.06) | 18.59 (0.03) | 18.54 (0.08) |
