# Supplementary material for: Pyruvate Oxidase as a Critical Link between Metabolism and Capsule Biosynthesis in Streptococcus pneumoniae
Source: PLoS Pathog. 2016 Oct 19;12(10):e1005951. doi: 10.1371/journal.ppat.1005951 (PMC5070856; doi:10.1371/journal.ppat.1005951)
Supplement: S3 Table — (DOCX) [file ppat.1005951.s003.docx]

**S3 Table. Median Log values of bacterial burden.**

**Strain Blood**  **Nasopharynx**

|  | **24 h** | **48 h** | **24 h** | **48 h** |
| --- | --- | --- | --- | --- |
| **T4** | 4.29 | 4.87 | 4.90 | 5.28 |
| **T4 *spxB*^-^** | --- | --- | 3.70 | 3.11 |
| **T4 *lctO*^-^** | 4.60 | 5.69 | 4.00 | 6.04 |
| **T4 *spxB*^-^ *lctO*^-^** | 4.41 | 5.22 | 5.48 | 4.95 |
| **T4 *lctO*^-/+^** | 4.51 | 4.81 | 5.51 | 5.78 |
| **T4 *spxB*^-^ *lctO*^-/+^** | --- | --- | 2.30 | 2.30 |
|  |  |  |  |  |
| **D39** | 6.00 | 7.46 | 4.74 | 4.02 |
| **D39 *spxB*^-^** | 3.70 | 6.76 | 3.52 | 3.01 |
| **D39 *lctO*^-^** | 3.48 | 5.42 | 4.26 | 3.17 |
| **D39 *spxB*^-^ *lctO*^-^** | 3.85 | 6.34 | 4.18 | 3.20 |
