# Supplementary material for: Pyruvate Oxidase as a Critical Link between Metabolism and Capsule Biosynthesis in Streptococcus pneumoniae
Source: PLoS Pathog. 2016 Oct 19;12(10):e1005951. doi: 10.1371/journal.ppat.1005951 (PMC5070856; doi:10.1371/journal.ppat.1005951)
Supplement: S4 Table — (DOCX) [file ppat.1005951.s004.docx]

**S4 Table. Strains used in this study.**

| Strain | Description | Source |
| --- | --- | --- |
| TIGR4 | TIGR4 wild type |  |
| TIGR4 *spxB*^-^ | *spxB* mutant in TIGR4; Erm^R^ | This study |
| TIGR4 *lctO*^-^ | *lctO* mutant in TIGR4; Spec^R^ | This study |
| TIGR4 *spxB*^-^ *lctO*^-^ | *spxB* and *lctO* double mutant in TIGR4; Erm^R^ Spec^R^ | This study |
| TIGR4 *pdhc*^-^ | *pdhc* mutant in TIGR4; Spec^R^ | This study |
| TIGR4R | Unencapsulated variant of TIGR4; Cm^R^ |  |
| D39 | D39 wild type |  |
| D39 *spxB*^-^ | *spxB* mutant in D39; Erm^R^ | This study |
| D39 *lctO*^-^ | *lctO* mutant in D39; Spec^R^ | This study |
| D39 *spxB*^-^ *lctO*^-^ | *spxB* and *lctO* double mutant in D39; Erm^R^ Spec^R^ | This study |
| D39 R6 | Unencapsulated variant of D39 |  |
| TIGR4::SweetJanus | SweetJanus replacement of capsule locus in TIGR4; Kan^R^ | This study |
| TIGR4::D39 | D39 capsule swapped in TIGR4::SweetJanus | This study |
| TIGR4::D39 *spxB*^-^ | *spxB* mutant in TIGR4::D39; Erm^R^ | This study |
| ABCA69 | Strain ABCA69; 4 serotype | CDC |
| ABCA69 *spxB*^-^ | *spxB* mutant in strain ABCA69; 4 serotype; Erm^R^ | This study |
| ABCB20 | Strain ABCB20; 4 serotype | CDC |
| ABCB20 *spxB*^-^ | *spxB* mutant in strain ABCB20; 4 serotype; Erm^R^ | This study |
| ABCB54 | Strain ABCB54; 4 serotype | CDC |
| ABCB54 *spxB*^-^ | *spxB* mutant in strain ABCB54; 4 serotype; Erm^R^ | This study |
| 6B | Strain CDC007; 6B serotype | CDC |
| 6B *spxB*^-^ | *spxB* mutant in strain CDC007; 6B serotype; Erm^R^ | This study |
| 12F | Strain CDC030; 12F serotype | CDC |
| 12F *spxB*^-^ | *spxB* mutant in strain CDC030; 12F serotype; Erm^R^ | This study |
| 18C | Strain CDC048; 18C serotype | CDC |
| 18C *spxB*^-^ | *spxB* mutant in strain CDC048; 18C serotype; Erm^R^ | This study |
| 35B | Strain ABCA31; 35B serotype | CDC |
| 35B *spxB*^-^ | *spxB* mutant in strain ABCA31; 35B serotype; Erm^R^ | This study |
| 45 | Strain Sp128; 45 serotype | CDC |
