# Supplementary material for: Pyruvate Oxidase as a Critical Link between Metabolism and Capsule Biosynthesis in Streptococcus pneumoniae
Source: PLoS Pathog. 2016 Oct 19;12(10):e1005951. doi: 10.1371/journal.ppat.1005951 (PMC5070856; doi:10.1371/journal.ppat.1005951)
Supplement: S5 Table — (DOCX) [file ppat.1005951.s005.docx]

**S5 Table. Primers used in this study.**

| **Primer Name** | **Sequence** |
| --- | --- |
| *Mutant Primers* |  |
| SpxB_Up_F | GCGAGCGAGTGAAGCTGG |
| SpxB_Up_R | GTTTGCTTCTAAGTCTTATTTCC-TTACTATTGAGAGGAAGTTAAAAAAATTTGAACC |
| SpxB_Down_F | GAGTCGCTTTTGTAAATTTGG-TTCCTCTCGCCGAAAATCAAATATGAAAC |
| SpxB_Down_R | CCATAGTCACTATATACGAGAATTTCGC |
| Erm_F | GGAAATAAGACTTAGAAGCAAAC |
| Erm_R | CCAAATTTACAAAAGCGACTC |
| LctO_Up_F | CGTGTTCATATGGTGGATGCTATC |
| LctO_Up_R | ATTATAACATGTATTCACGAACGAAAATCGAT-AAACTGTCCTCCTCGATTAAGTAAG |
| LctO_Down_F | gattttagaaaacaataaacccttgcatatg-TAAAACAGATTGCCTCCACTGAATGT |
| LctO_Down_R | CTGATGAAGCCACCGCCG |
| Pdhc_Up_F | CTGGTGCTGGTTTAGGAACATCC |
| Pdhc_Up_R | CATGTATTCACGAACGAAAATCGAT-CTTCTTGCCTTTCTAACTTTCTTCTTACC |
| Pdhc_Down_F | AGAAAACAATAAACCCTTGCATATG-GGTTACATAAGATAAGTTATGTAAATA |
| Pdhc_Down_R | ATCGGACCAAAGGACTGAACACC |
| Spec_F | atcgattttcgttcgtgaatac |
| Spec_R | CATATGCAAGGGTTTATTGTTTTC |
|  |  |
| *qRT-PCR Primers* |  |
| Cps4A_F | GCCTTGGTAGGGCTACTCTTG |
| Cps4A_R | CTGCAAAGAGCGACACAGAG |
| Cps4E_F | CCTAGACCAGCGGGTATAAATG |
| Cps4E_R | GTAGCCATCTAACTCCGTCTTC |
| MnaA_F | GAACTCGTCCAGAAGCCATC |
| MnaA_R | CCAACATTTGACGGTGTTGGC |
| FnlC_F | GACACTCCGGACGTATACATGG |
| FnlC_R | CGCTTGGCAGCAATAGCTG |
| SpxB_F | CTACGGTATCCCATCAGGAAC |
| SpxB_R | GCACCTGTCTCTTCGTGG |
| LctO_F | GCTCATCGTTCCTCATACAC |
| LctO_R | CTCTGGAAGGTCAACAGTAGAG |
| GyrA_F | CCGTGACGGGTTGTAAAGAAGAGATAAG |
| GyrA_R | AGCACCCATGATCATCTGCTCTTCTTCAC |
| SodA_F | TGCATACGACGCTTTGGAACC |
| SodA_R | GCTGCATTGGCATTGTTGAC |
| TpxD_F | AACTACAAGTCGGCGACAAGG |
| TpxD_R | CGATAGAAGGAACGACACTCAAG |
| ertX1_F | ATGGCAAACATGTGTTCTTGGAG |
| erX1_R | CTAGCTGTCTGCTCCGTCTTCAT |
