# Supplementary material for: Pyruvate Oxidase as a Critical Link between Metabolism and Capsule Biosynthesis in Streptococcus pneumoniae
Source: PLoS Pathog. 2016 Oct 19;12(10):e1005951. doi: 10.1371/journal.ppat.1005951 (PMC5070856; doi:10.1371/journal.ppat.1005951)
Supplement: S1 Fig — Hydrogen peroxide production was determined in two methods. Strains include wild type and the spxB, lctO, and the spxB lctO double mutant in the TIGR4 (A, C, F) and D39 (B, D, F) backgrounds, as well as the TIGR4 lctO mutant and double mutant complemented with pABG5-lctO (lctO -/+ and spxB - lctO -/+) (A, C), the TIGR4 pdhc mutant (E), and the wild type and spxB mutant in the TIGR4 with D39 capsule (F). Production by colonies on plates was indicated by purple coloration (A, B). Strains were serially diluted 1:10 from left to right. Production in cell culture was determined using the Amplex Red kit (C, D, E, F). Values were normalized to total cell protein and then plotted as percentage of wild type, where TIGR4 produces 1.87 ± 0.27 μM/μg cellular protein and D39 produces 2.11 ±1.08 μM/μg cellular protein. Hydrogen peroxide production was compared using unpaired parametric t test; all mutants compared to wild type were significant with p<0.001. (DOCX) [file ppat.1005951.s008.docx]

**
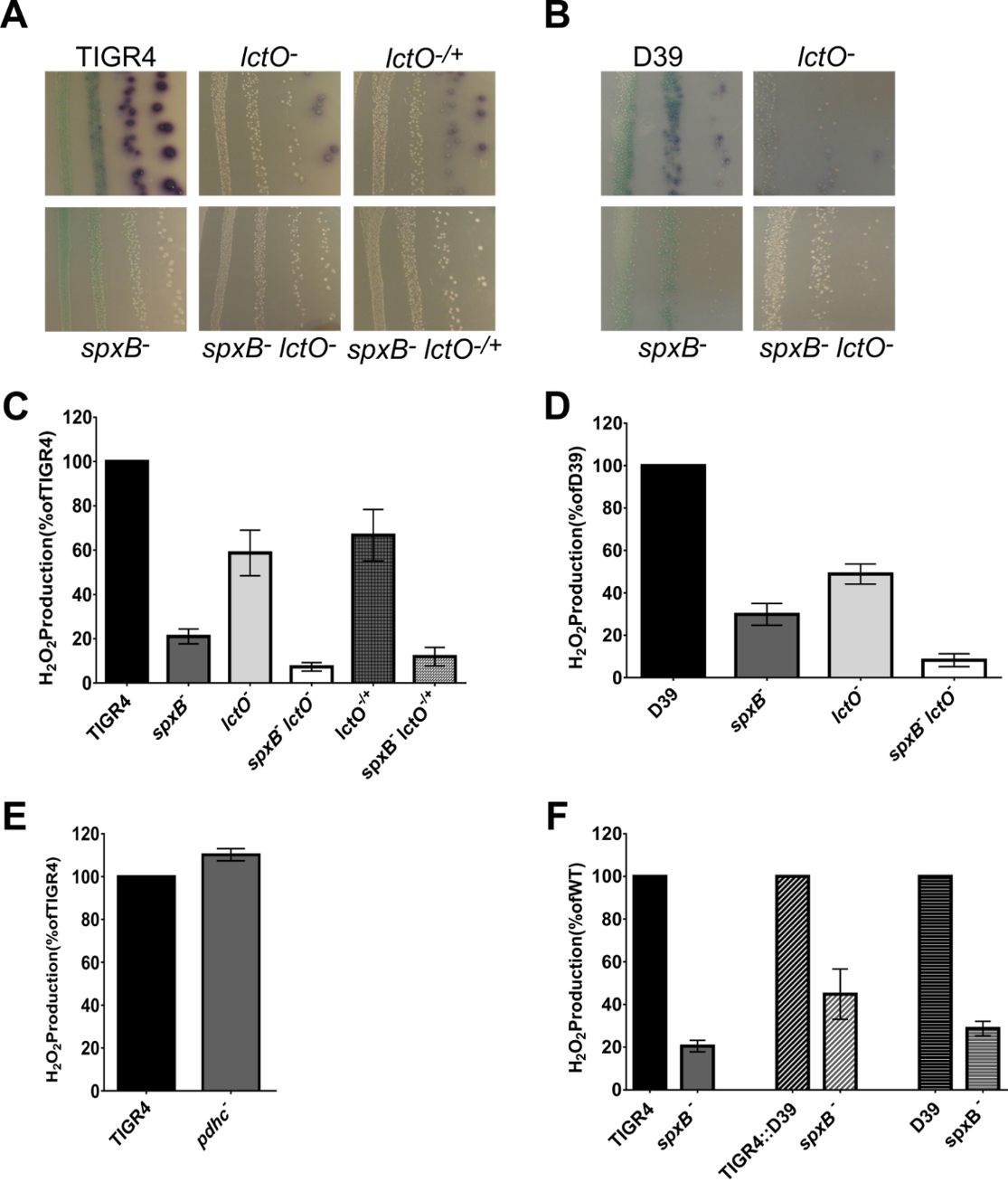
**

**S1 Fig. Hydrogen peroxide production is reduced in the *spxB* mutant and is negligible in the double mutant in TIGR4 and D39.** Hydrogen peroxide production was determined in two methods. Strains include wild type and the *spxB*, *lctO*, and the *spxB* *lctO* double mutant in the TIGR4 (**A**, **C, F**) and D39 (**B**, **D, F**) backgrounds, as well as the TIGR4 *lctO* mutant and double mutant complemented with pABG5-lctO (*lctO^-/+^* and *spxB^-^lctO^-/+^*) (**A, C**), the TIGR4 *pdhc* mutant (**E**), and the wild type and *spxB* mutant in the TIGR4 with D39 capsule (**F**). Production by colonies on plates was indicated by purple coloration (**A**, **B**). Strains were serially diluted 1:10 from left to right. Production in cell culture was determined using the Amplex Red kit (**C**, **D, E, F**). Values were normalized to total cell protein and then plotted as percentage of wild type, where TIGR4 produces 1.87 ± 0.27 µM/µg cellular protein and D39 produces 2.11 ±1.08 µM/µg cellular protein. Hydrogen peroxide production was compared using unpaired parametric t test; all mutants compared to wild type were significant with p<0.001.
