# Supplementary material for: Pyruvate Oxidase as a Critical Link between Metabolism and Capsule Biosynthesis in Streptococcus pneumoniae
Source: PLoS Pathog. 2016 Oct 19;12(10):e1005951. doi: 10.1371/journal.ppat.1005951 (PMC5070856; doi:10.1371/journal.ppat.1005951)
Supplement: S2 Fig — Functional complementation with pABG5-lctO was determined. Strains include TIGR4 and the spxB, lctO, and spxB lctO mutants, and the lctO mutant and double mutant complemented with pABG5-lctO (lctO -/+ and spxB - lctO -/+). Capsule production was measured using capsule blotting method (A). BALB/c mice were infected IN with 1 x 10^7^ cells and monitored for disease progression (B-F). Survival of mice was followed for 8 days (B). For clarity purposes, the survival curves of the spxB - lctO -/+ mutant was nudged. Bacterial presence in the blood was determined at 24 hours (C) and 48 hours (D) post infection. In the same mice, bacterial carriage in the nasopharynx was determined at 24 hours (E) and 48 hours (F) post infection. The titer and survival data for the wild type and mutants was used in conjunction with other mouse studies in Figs 4 and 5 and are included here for comparison with the results from mice infected with the complemented strains, which were performed at the same time. Survival data were analyzed using the Mantel-Cox log rank test. p = 0.0018 for spxB mutant and the complemented double mutant compared to wild type; p = 0.0394 for the double mutant compared to wild type; the lctO mutant and complement compared to the wild type were non-significant; the complemented double mutant compared to the spxB mutant was non-significant. For blood titers, mutant strains were compared to wild type using nonparametric Mann-Whitney t test; * p = 0.05–0.01, ** p = 0.01–0.001. (DOCX) [file ppat.1005951.s009.docx]

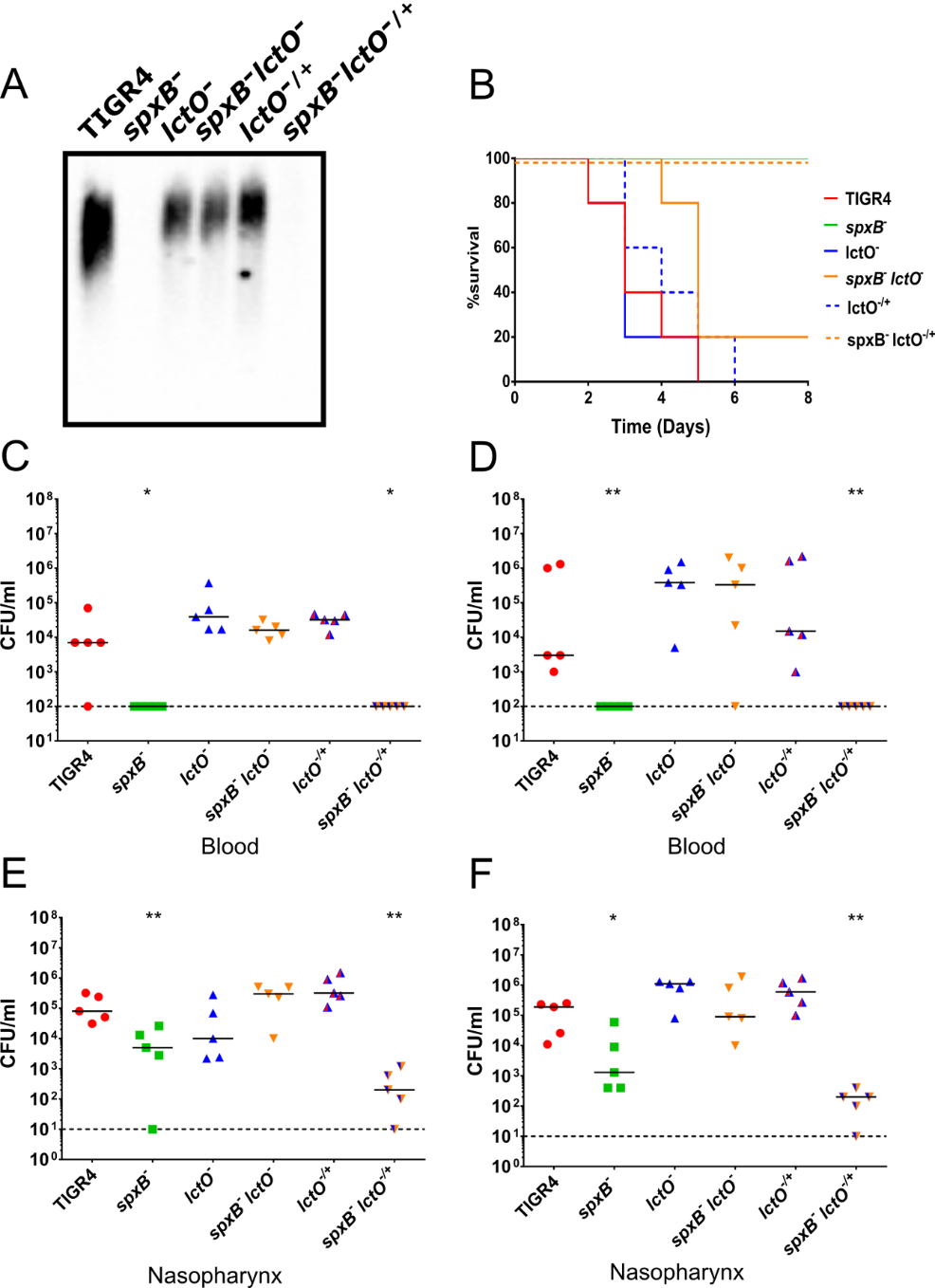


**S2 Fig. Complementation of *lctO* to the double mutant restores the *spxB* mutant phenotype.** Functional complementation with pABG5-lctO was determined. Strains include TIGR4 and the *spxB*, *lctO*, and *spxB* *lctO* mutants, and the *lctO* mutant and double mutant complemented with pABG5-lctO (*lctO^-/+^* and *spxB^-^lctO^-/+^*). Capsule production was measured using capsule blotting method (**A**). BALB/c mice were infected IN with 1 x 10^7^ cells and monitored for disease progression (**B-F**). Survival of mice was followed for 8 days (**B**). For clarity purposes, the survival curves of the *spxB^-^lctO^-/+^* mutant was nudged. Bacterial presence in the blood was determined at 24 hours (**C**) and 48 hours (**D**) post infection. In the same mice, bacterial carriage in the nasopharynx was determined at 24 hours (**E**) and 48 hours (**F**) post infection. The titer and survival data for the wild type and mutants was used in conjunction with other mouse studies in Figures 4 and 5 and are included here for comparison with the results from mice infected with the complemented strains, which were performed at the same time. Survival data were analyzed using the Mantel-Cox log rank test. p=0.0018 for *spxB* mutant and the complemented double mutant compared to wild type; p=0.0394 for the double mutant compared to wild type; the *lctO* mutant and complement compared to the wild type were non-significant; the complemented double mutant compared to the *spxB* mutant was non-significant. For blood titers, mutant strains were compared to wild type using nonparametric Mann-Whitney t test; * p=0.05-0.01, ** p=0.01-0.001.
