# Supplementary material for: Pyruvate Oxidase as a Critical Link between Metabolism and Capsule Biosynthesis in Streptococcus pneumoniae
Source: PLoS Pathog. 2016 Oct 19;12(10):e1005951. doi: 10.1371/journal.ppat.1005951 (PMC5070856; doi:10.1371/journal.ppat.1005951)
Supplement: S3 Fig — Intracellular steady-state levels of ATP (A) and acetyl-phosphate (B) were determined. Strains include TIGR4 and the spxB, lctO, and spxB lctO mutants. Values were normalized to total cell protein and then plotted as percentage of wild type. ATP and acetyl-phosphate levels were compared using unpaired parametric t test; **, p = 0.01–0.001; ***, p<0.001. (DOCX) [file ppat.1005951.s010.docx]

**S3 Fig. ATP and acetyl-phosphate steady-state levels are reduced in the *spxB* and double mutants.** Intracellular steady-state levels of ATP (**A**) and acetyl-phosphate (**B**) were determined. Strains include TIGR4 and the *spxB*, *lctO*, and *spxB* *lctO* mutants. Values were normalized to total cell protein and then plotted as percentage of wild type. ATP and acetyl-phosphate levels were compared using unpaired parametric t test; **, p=0.01-0.001; ***, p<0.001.
